# Insights into Electrophilic Methane Activation with High-Valent Lead in Trifluoroacetic Acid and Trifluoromethanesulfonic Acid

**DOI:** 10.1021/acsomega.6c06786

**Published:** 2026-08-11

**Authors:** Liam A. Parkin, Andrew J. P. White, Andrew E. Ashley, George J. P. Britovsek

**Affiliations:** Department of Chemistry, 4615 Imperial College London, Molecular Sciences Research Hub, White City Campus, 82 Wood Lane, London W12 0BZ, U.K.

## Abstract

The formation of high-valent lead compounds has been investigated in trifluoroacetic acid (HTFA) and trifluoromethyl sulfonic acid (HOTf). Pb­(IV) species are formed in these acids, starting from Pb_3_O_4_ or other Pb­(IV) precursors (Pb­(OAc)_4_, PbPh_4_). Attempts to isolate Pb­(IV) compounds resulted in the isolation of the Pb­(II) salts Pb­(TFA)_2_(HTFA)_2_ and Pb­(OTf)_2_(HOTf), which have been structurally characterized. Pb­(TFA)_4_ and Pb­(OTf)_4_ can undergo reductive elimination in solution to give their respective Pb­(II) compounds and formation of CF_3_TFA and CF_3_OTf, together with loss of CO_2_ and SO_3_, respectively. A solution of Pb­(OTf)_4_ in HOTf/Tf_2_O activates methane at 5 bar at 60 °C to give MeTf, MeOTf and HOTf. The formation of PbMe­(OTf)_3_ is believed to be a key intermediate in these reactions, which reacts with Tf_2_O to form MeTf and Pb­(OTf)_4_, and undergoes reductive elimination to form MeOTf and Pb­(OTf)_2_.

## Graphical abstract

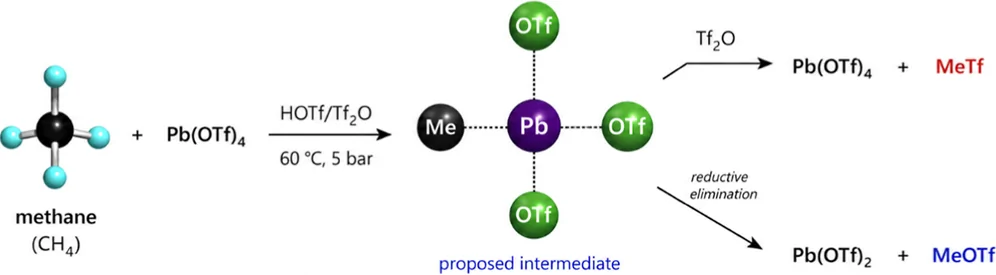

## Introduction

The selective functionalization of small alkanes remains one of the great challenges in catalysis research.^1^ The abundant availability of methane and other light alkanes from shale gas has led to an increase in natural gas conversion in recent years.^2^ Methane is predominantly converted to syngas via steam reforming, whereas higher alkanes are generally converted to C2/C3 olefins, for example ethane to ethylene via steam cracking or propane to propylene by dehydrogenation.^3^

Considerable progress has been made during the last three decades regarding the organometallic activation of alkanes in solution, in particular through electrophilic activation with late transition metals.^1,4–12^ This method results in the oxidation of methane to methanol (or a methanol ester) together with the reduction of the metal center, which will require reoxidation in order to achieve catalytic turnover. Both chemical oxidants, for example SO_3_,^13–15^ and electrochemical approaches have been reported for this reoxidation.^16,17^

The application of high valent main group metals for alkane activation is much less developed compared to transition metal systems.^1,18^ Main group compounds in high oxidation states based on the heavier elements are highly electrophilic and can perform C–H activation of hydrocarbons, similar to transition metal systems. Examples can be found across the various groups of the p-block elements, including Tl, Pb, Sb and I.^19–22^ For Group 14 metals, it was shown more than 50 years ago that Pb­(TFA)_4_ (TFA = trifluoroacetate, CF_3_CO_2_^–^) is able to activate benzene and heptane at room temperature to the corresponding esters,^23–25^ as well as other hydrocarbons.^26–28^ Subsequently, Pb­(TFA)_4_ and also Tl­(TFA)_3_ were shown to activate light alkanes, including methane.^4,19^ These reactions were carried out in trifluoroacetic acid (HTFA) at 35 bar and 180 °C generating MeTFA, a reduced lead compound Pb­(TFA)_2_ and one equivalent TFAH, as shown in eq 1. The ester product MeTFA is protected from overoxidation by the electron withdrawing trifluoroacetate group. More recently, Neumann and co-workers have shown that in situ reoxidation of Pb­(II) to Pb­(IV) can be achieved using electrochemical methods in HTFA, making this process potentially catalytic.^29^

1 $$Pb{\left(TFA\right)}_{4}+{CH}_{4}\begin{array}{l} TFAH\\ \to \\ 180^{{}^{\circ}}C,35bar \end{array}Pb{\left(TFA\right)}_{2}+H_{3}CTFA+TFAH$$

The reaction in eq 1 relies on the C–H activation ability of high valent main group compounds. Comparable C–H activation chemistry is also seen in transition metal systems, but methane coordination is inhibited due to solvent coordination to the metal center. The lack of ligand field stabilization in main group metal complexes means solvents are coordinatively labile and methane coordination is comparatively facile, even when the metal is in its highest oxidation state and thus highly electrophilic.^9,19^ The +4 oxidation state of Group 14 elements becomes increasingly less stable down the group due to the inert pair effect, as seen for example in the trifluoroacetate compounds in this group. Si­(TFA)_4_ is a stable liquid whereas Ge­(TFA)_4_ decomposes slowly at room temperature. Sn­(TFA)_4_ is a relatively stable solid, but yields are generally low due to the elimination of trifluoroacetic anhydride and formation of stannoxanes.^30^ To the best of our knowledge, Pb­(TFA)_4_ has never been isolated and convincingly characterized.^31^ In general, Pb­(IV) compounds with electron-withdrawing substituents are unstable at room temperature, for example PbCl_4_ readily forms PbCl_2_ and Cl_2_.^32^ Electron donating ligands such as alkyls or additional donors can provide some stabilization,^33^ hence PbMe_4_ and PbEt_4_ and various mixed R_*n*_PbX_4–*n*_ are relatively stable compounds,^34^ and Pb­(OAc)_4_ is a stable solid featuring an eight-coordinated Pb­(IV) center with four bidentate acetate ligands.^35^

In this study, our rationale has been that the rate-determining step for methane conversion with high valent main group compounds is C–H bond activation, once methane is coordinated to the Pb­(IV) center. An enhancement in electrophilicity of the metal center should thus result in greater activating ability due to increased acidification of the C–H bond.^36^ Ligands that are more electron-withdrawing should thus enhance electrophilicity and triflate anions (OTf^–^, CF_3_SO_3_^–^) are exceptional in this respect as they are more electron-withdrawing than trifluoroacetate, and are oxidatively very stable, up to +3 V (vs NHE).^37,38^ Pb­(OTf)_4_ was reportedly obtained from Pb­(TFA)_4_, but in light of the results shown below, the characterization of this product by elemental analysis only is not reliable in our view.^31^ Other early attempts to prepare Pb­(OTf)_4_ have shown that the addition of HOTf to PbR_4_ compounds leads to R_3_Pb­(OTf) and an excess of HOTf only leads to R_2_Pb­(OTf)_2_, as RPb­(OTf)_3_ compounds are believed to be too unstable to be isolable.^39^

Here we have investigated the synthesis and characterization of lead trifluoroacetate and triflate compounds. Both Pb­(II) and Pb­(IV) compounds have been targeted, the latter being highly electrophilic and reacting readily with hydrocarbons, including methane. We have found that high valent and highly electrophilic Pb­(OTf)_4_ shows facile methane activation under mild conditions.

## Results and Discussion

### Synthesis and Characterization of Lead­(II) Trifluoroacetate

The preparation of Pb­(TFA)_4_ was first reported by Convery in 1960 via the acidolysis of red lead (Pb_3_O_4_) in trifluoroacetic acid (HTFA) and trifluoroacetic anhydride (TFAA) according to eq 2.^40^ In their work, upon cooling of the reaction mixture to 0 °C, a white crystalline product was isolated, which was assumed to be Pb­(TFA)_4_, although no analytical data was provided. Sternhell later reported a similar synthetic procedure, but separated the solid and liquid phase at the end of the reaction,^41^ and only the liquid phase was used for further reactions of Pb­(TFA)_4_ in TFAH, whereby the Pb­(IV) concentration was determined by iodometry.

2 $${Pb}_{3}O_{4}+8TFAH\begin{array}{l} TFAH/TFAA\\ \to \\ 2-4days,RT \end{array}Pb{\left(TFA\right)}_{4}+2Pb{\left(TFA\right)}_{2}+4H_{2}O$$

We carried out the reaction as shown in eq 2 as per Convery’s method and at the end of the reaction, the solution was filtered and the filtrate concentrated. Upon cooling, large colorless crystals were isolated, which were identified by scXRD as the Pb­(II) compound Pb­(TFA)_2_(HTFA)_2_ (Figure 1).

**Figure 1:**
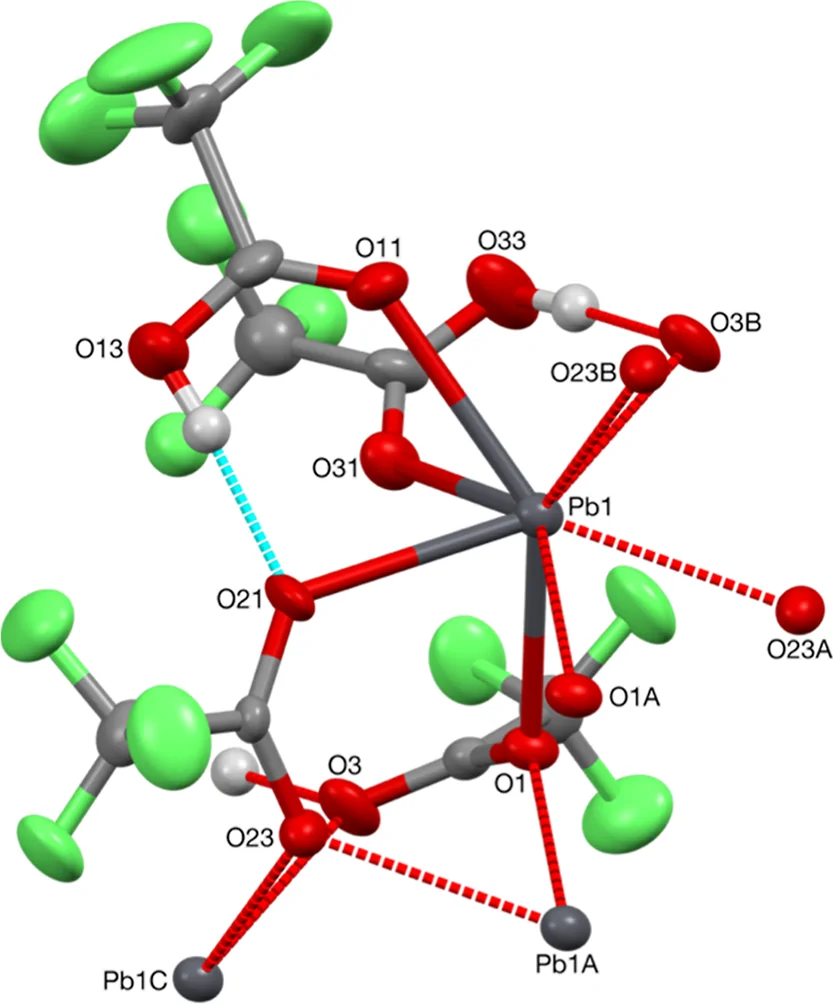
Crystal structure of Pb­(TFA)_2_(HTFA)_2_ showing the asymmetric unit and how it links to adjacent atoms (50% probability ellipsoids). Pb (dark gray), C (medium gray), O (red), F (green), H (light gray).

Initially we erroneously assumed that the structure was Pb­(TFA)_4_, but closer inspection of the structure revealed some anomalies in the Pb–O distances, which could be explained invoking the presence of two additional protons and the compound was therefore determined as Pb­(TFA)_2_(HTFA)_2_, i.e. Pb­(II) rather than Pb­(IV). Pb­(TFA)_2_(HTFA)_2_ is the first structurally characterized example of a homoleptic lead trifluoroacetate compound. The structure is a polymeric chain, extending along the a direction. In each asymmetric unit, the Pb­(II) centers are connected by bridging trifluoroacetate groups and two TFAH ligands are terminally bound (connected to O11 and O31). The two TFA^–^ ligands are bridging (connected to O1 and O21) with secondary bonds to another lead atom by the carbonyl oxygen on each (O3 and O23 respectively). The Pb­(II) centers have a coordination number of 8 with near square antiprismatic geometry. The extended structure of Pb­(TFA)_2_ differs considerably from molecular compounds such as Pb­(OAc)_2_ and Pb­(OAc)_4_, where the acetate ligands act as bidentate ligands coordinating to the same Pb center.^35,42^ The stronger coordination of the trifluoroacetate ligands to Pb­(II) in Pb­(TFA)_2_ is reflected in the smaller Pb–O bond lengths, which are on average 0.1 Å shorter compared to Pb­(OAc)_2_. ^19^F NMR analysis in CDCl_3_ provides the trifluoroacetate signal at −75 ppm. Attempts were made to measure ^207^Pb NMR spectra in solution at room temperature, but no signal was observed, presumably due to fast ligand exchange.

### Synthesis of Lead­(II) and Lead­(IV) Trifluoromethanesulfonate

The reaction of the trifluoroacetate salt Pb­(TFA)_2_(HTFA)_2_ with HOTf followed by removal of TFAH by distillation and crystallization from a mixture of triflic acid/chloroform resulted in the precipitation of crystals suitable for scXRD. The structure obtained corresponds to the Pb­(II) compound Pb­(OTf)_2_(HOTf) (See Figure 2 for Pb­(OTf)_2_(HOTf)-A). In the asymmetric unit for Pb­(OTf)_2_(HOTf), the central lead is surrounded by three triflate groups. The extended structure is a linear coordination polymer. The polymer consists of bridging triflate groups, with each monomer unit consisting of four terminal triflate groups (only two of the three triflate oxygens bind to lead centers), and two fully bridging triflates (where all of the oxygens coordinate to a Pb center). Each Pb­(II) center has a coordination number of 8 with a slightly distorted square antiprismatic geometry. The structure of Pb­(OTf)_2_(HOTf) is somewhat related to Pb­(OTf)_2_(H_2_O), the only other structurally characterized example of a homoleptic lead­(II) triflate compound,^43^ which was prepared from PbO and HOTf.^44^ Another preparation of Pb­(OTf)_2_ has been reported from PbCl_2_ with an excess HOTf.^45^

**Figure 2:**
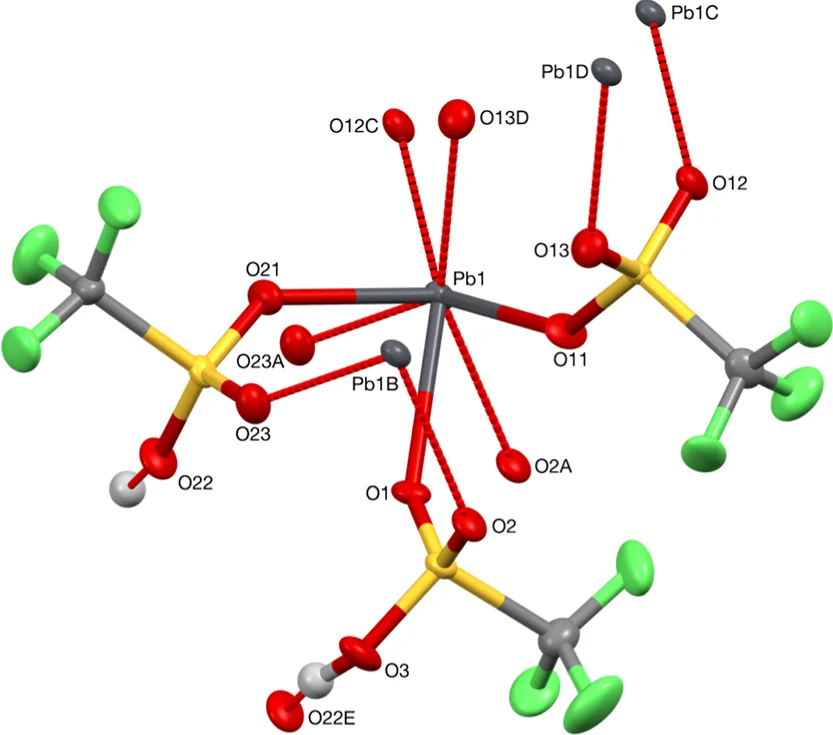
Crystal structure of Pb­(OTf)_2_(HOTf)-A showing the asymmetric unit and how it links to adjacent atoms (50% probability ellipsoids). Pb (dark gray), C (medium gray), O (red), F (green), S (yellow), H (light gray).

Several Pb­(IV)-containing starting materials were investigated in our attempts to prepare Pb­(OTf)_4_, including Pb_3_O_4_, Pb­(OAc)_4_ and PbPh_4_. These attempts resulted in several crystalline products and additional X-ray structures, Pb­(OTf)_2_(HOTf)-B from Pb_3_O_4_ and Pb­(OTf)_2_(HOTf)-C from Pb­(OAc)_4_, all with the same unit cell, confirming the formation of Pb­(OTf)_2_(HOTf) in all cases (see Supporting Information). PbO_2_ showed no reaction with HOTf at room temperature. Higher temperatures are generally required for the reaction of PbO_2_ with acids HX (X = Cl^–^, NO_3_^–^) resulting in reduction to PbX_2_ and the formation of O_2_. The formation of reduced Pb­(II) compounds from Pb­(IV) precursors requires something else to be oxidized. When Pb­(OAc)_4_ was dissolved in HOTf, the formation of MeOTf was observed by ^1^H NMR spectroscopy (see Figure S1), presumably via decarboxylation of the peroxo species AcOOTf formed through reductive elimination (eq 3). AcOOTf has not been reported, but the related compound AcOTFA is known to be unstable and decarboxylates to give MeTFA.^46^ A similar decarboxylation of AcOOTf could therefore have resulted in MeOTf.

3 $$Pb{\left(OAc\right)}_{4}+HOTf{\underset{\begin{array}{l} -HOAC\\ -Pb{\left(OAc\right)}_{2} \end{array}}{\to }}^{\prime \prime }{ACOOTf}^{\prime \prime }\underset{-{CO}_{2}}{\to }MeOTf$$

The product from the reductive elimination of Pb­(OTf)_4_ to Pb­(OTf)_2_ would be the peroxide TfOOTf, which is known but unstable above 10 °C and decomposes to give SO_3_ and the trifluoromethyl triflate ester F_3_CO_3_SCF_3_ (F_3_COTf) (eq 4).^47,48^ This product was indeed observed in the ^19^F NMR spectrum of the reaction between Pb_3_O_4_ in HOTf with characteristic signals at −54.5 and −75.1 ppm and ^5^ *J*_FF_ = 3.5 Hz (see Figure S2). These observations suggest that Pb­(OTf)_4_ is formed in situ, but slowly decomposes to Pb­(OTf)_2_ according to eq 4. The SO_3_ generated in this process is likely to react with HOTf in situ to generate the disulfonic acid F_3_CS_2_O_6_H.^48^

4

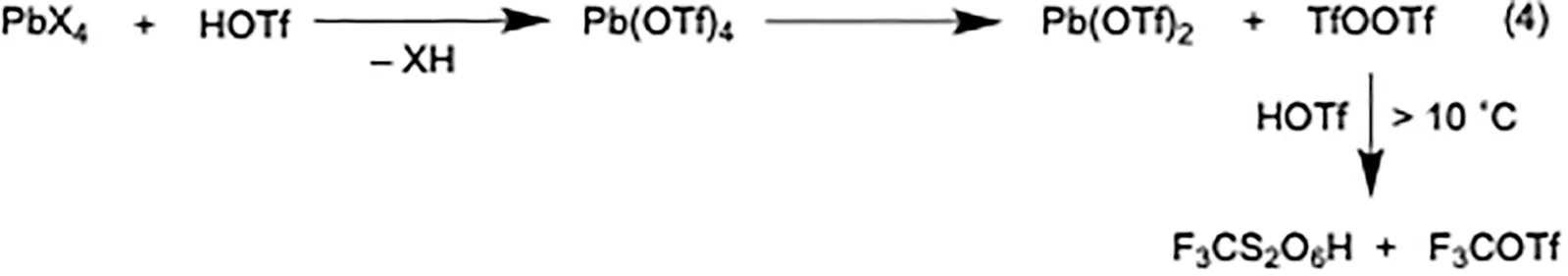




#### Electrochemical Studies

Electrochemical oxidation of Pb­(OTf)_2_ has been carried out in triflic acid at room temperature with NaOTf as the electrolyte, a glassy carbon working electrode, Pt wire as the counter electrode and Ag wire as the reference electrode.^38^ A solution of Pb­(OTf)_2_, prepared from PbCl_2_,^45^ in HOTf shows an irreversible oxidation at +1.53 V (vs Ag) (Figure 3). A solution of [Ru­(bipy)_3_]­Cl_2_ was used as an external standard, which showed a reversible Ru^2+/3+^ redox couple at +0.31 V (vs Ag), similar to previously reported values (Figure S8).^38^ Considering the [Ru­(bipy)_3_]^2+/3+^ couple occurs at +1.26 V vs SHE, this would place the Pb^2+/4+^ couple for Pb­(OTf)_2_ in HOTf at approximately +2.48 V vs SHE. This oxidation potential is significantly higher than other reported values for Pb^2+/4+^ redox couples, for example the Pb^2+/4+^ couple for Pb­(NO_3_)_2_ in aqueous solution is reported at +1.2 V vs SCE (+1.5 V vs SHE),^49^ and in H_2_SO_4_ (0.5M) at +1.80 V vs SHE.^50^ In nonaqueous solvents, Pb­(OAc)_2_ in CH_3_CN was determined at +1.60 V vs Ag/AgCl (+1.80 V vs SHE),^51^ and Pb­(benzoate)_2_ in CH_3_CN at +1.60 V vs SCE (+1.85 V vs SHE).^52^ The onset of oxidation for Pb­(TFA)_2_ in HTFA was reported at approximately ∼1 V (vs SHE), although a potential of +1.87 V was used for efficient oxidation of Pb^2+/4+^.^29^ Also noteworthy, the electrocatalytic oxidation of methane at PbO_2_-coated electrodes in alkaline solution was reported to require +2.1 V (vs RHE).^53^ The high oxidation potential for Pb^2+/4+^ seen here for Pb­(OTf)_2_ in triflic acid solution, suggests Pb­(OTf)_4_ should be a very powerful oxidant and enable methane oxidation under relatively mild conditions.

**Figure 3:**
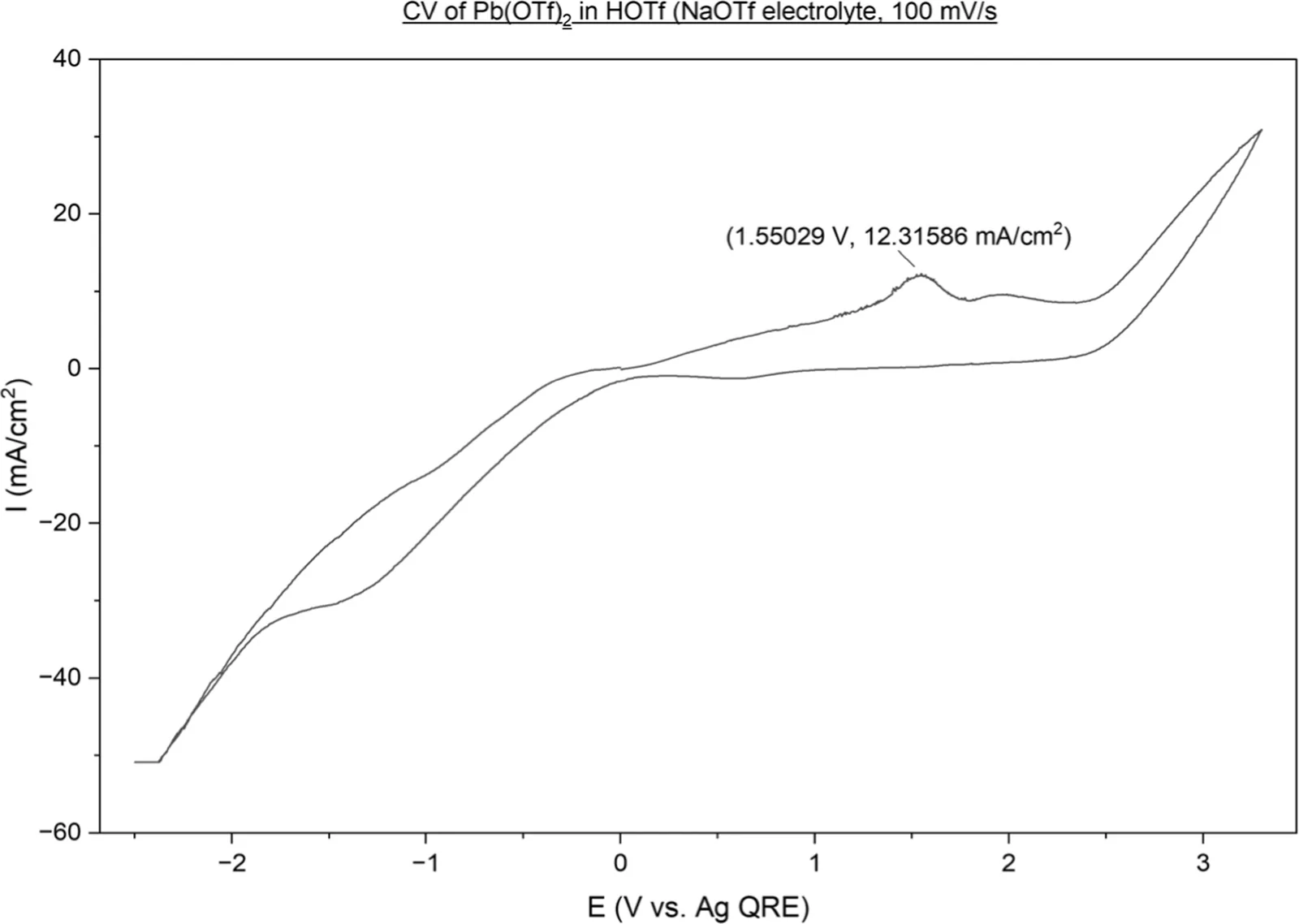
Cyclic voltammogram of Pb­(OTf)_2_ (0.5 mM) in HOTf with NaOTf (0.1 M) as electrolyte.

#### Methane Activation

Previous studies have shown that stoichiometric methane conversion can be achieved with Pb­(TFA)_4_ in HTFA at 180 °C and 35 bar CH_4_ pressure to give MeTFA in 76% yield after 3 h.^19^ Neumann and co-workers showed that catalytic turnover of methane to MeTFA is possible at 30 bar pressure and room temperature under electrocatalytic conditions, resulting in an estimated TOF of 50 min^–1^ after 7 h.^29^

Here we have generated Pb­(OTf)_4_ in situ from Pb_3_O_4_ in HOTf (with the addition of a small amount of Tf_2_O to scavenge any moisture) at room temperature, to generate initially a 1:2 mixture of Pb­(IV) and Pb­(II) triflate (eq 5). Some decomposition of Pb­(IV) can occur during the preparation due to reductive elimination, as discussed previously, and the amount of active Pb­(IV) in the final product was determined by iodometry to be at least 33% of the expected amount (see Supporting Information).

5 $${Pb}_{3}O_{4}+8HOTf\begin{array}{l} HOTf/Tf_{2}O\\ \to \\ 2-4days,RT \end{array}Pb{\left(OTf\right)}_{4}+2Pb{\left(OTf\right)}_{2}+4H_{2}O$$

The solution was placed in a high-pressure NMR tube and after heating to 60 °C under a pressure of 5 bar CH_4_ two new signals are observed in the ^1^H NMR spectrum (Figure S3). One signal at 4.00 ppm is the expected product methyl triflate (MeOTf). The other signal at 3.04 ppm was identified as methyl triflone, CH_3_SO_2_CF_3_ (MeTf) with a characteristic coupling ^4^J_H–F_ = 0.9 Hz (Figures S4–S5).^54^ Conversions are generally low under these conditions, with a yield of 2% of MeOTf and 3.2% of MeTf after 15 h. No other products were detected. Experiments using CD_4_ instead of CH_4_ showed the formation of CD_3_SO_2_CF_3_ (CD_3_Tf) by ^2^H NMR, which confirms that this product is methane derived (Figure S6). No formation of CD_3_OTf was observed within 7 days, which indicates that the reaction with CD_4_ is noticeably slower, indicative of C–D/H activation being the rate-determining step.

It is observed that the formation of MeTf precedes the formation of MeOTf. This is believed to proceed through a pathway where Pb­(OTf)_4_ reacts with methane and C–H activation generates the Pb­(IV) intermediate MePb­(OTf)_3_ and HOTf. Monoalkyl (and aryl) Pb­(IV) compounds of type RPbX_3_, where X is an electron-withdrawing ligand, are generally unstable and undergo reductive elimination to form RX and PbX_2_.^33,55,56^ Here, the intermediate MePb­(OTf)_3_ is assumed to react first with the available Tf_2_O, which leads to MeTf (eq 6) and regenerates Pb­(OTf)_4_. Once most Tf_2_O has been consumed, reductive elimination of MePb­(OTf)_3_ proceeds to generate MeOTf and Pb­(OTf)_2_ (eq 6). The reaction of MePb­(OTf)_3_ with Tf_2_O to generate MeTf would be analogous to the known reactivity of Tf_2_O with other organometallic methyl compounds such as MeLi and Grignards.^54,57^


6

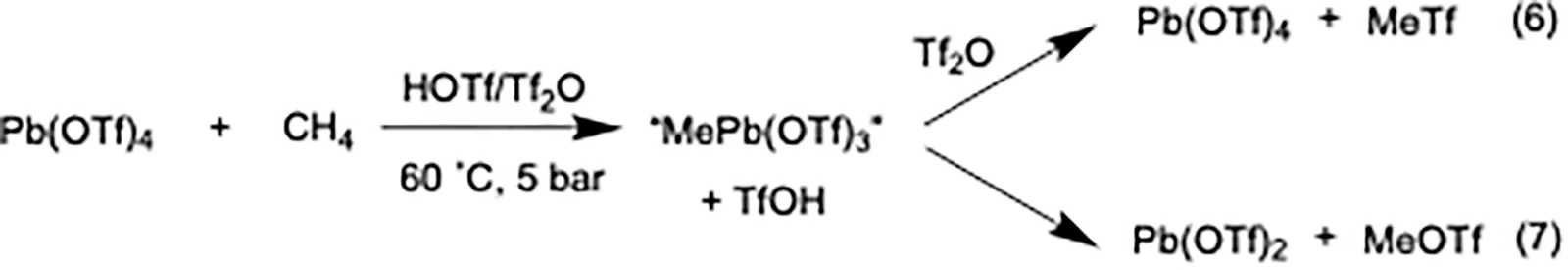




Alternative mechanisms have been considered, such as a radical-based mechanism akin to oxidation reactions reported by Kochi et al. using Pb­(OAc)_4_ and carboxylic acids, which leads to alkyl radicals after oxidation of the carboxylate anion and decarboxylation.^58^ In the case of Pb­(OTf)_4_ in triflic acid used here, the oxidation of triflate anions to triflate radicals would be followed by Hydrogen Atom Transfer (HAT) from methane to generate methyl radicals and eventually MeOTf. However, the absence of any products, other than MeTf and MeOTf, that could be expected from a radical-based mechanism such as C_2_H_6_, CF_3_H, C_2_F_6_, disfavor this mechanism. It is also not clear how MeTf could be generated via this radical mechanism. Methane activation reactions via HAT and free-radical mechanisms have also been reported in other acidic media such as oleum^59^ or H_2_SO_4_/TFAA (TFAA = trifluoroaceticanhydride),^60^ but these reactions generally yield methanesulfonic acid as the main product, which we have never observed here. Lastly, considering HOTf is a superacid (p*K*_a_ = −15), we also considered a possible carbocationic mechanism, like the work on alkane activation in superacidic media pioneered by Olah.^61^ Methane activation in FSO_3_H/SbF_5_ involves protonation and formation of CH_5_^+^ and CH_3_^+^ cations (and H_2_), which undergo polycondensation to give higher alkanes.^62^ Here in HOTf this could lead to MeOTf, but again it would not explain the formation of MeTf. There is no reaction of methane in HOTf in the absence of Pb under the conditions used (60 °C, 5 bar). We have also never seen the formation of H_2_ or any higher alkanes.

In conclusion, our attempts to isolate and characterize high valent Pb­(IV) compounds Pb­(TFA)_4_ and Pb­(OTf)_4_ have been unsuccessful so far, but these reactions have led to the isolation and first structural characterization of novel Pb­(II) compounds Pb­(TFA)_2_(HTFA)_2_ and Pb­(OTf)_2_(HOTf). The reduction of Pb­(IV) to Pb­(II) is believed to proceed via reductive elimination of TFA–TFA or TfO-OTf, which are prone to loss of CO_2_ and SO_3_ respectively and the formation of the respective trifluoromethyl esters CF_3_TFA and CF_3_OTf, the latter has been observed spectroscopically. High valent Pb­(IV) triflate can be generated in solution and we have demonstrated that in situ generated Pb­(OTf)_4_ is capable of methane activation to form MeOTf at 60 °C and 5 bar pressure. The reaction is assumed to proceeds through PbMe­(OTf)_3_ as an unstable intermediate. According to NMR analysis, this intermediate reacts with Tf_2_O to form MeTf and Pb­(OTf)_4_ and subsequently, after most Tf_2_O has been consumed, leads to reductive elimination to form MeOTf and Pb­(OTf)_2_. Reaction conditions for methane activation with Pb­(OTf)_4_ in HOTf are milder than those previously reported for the analogous reactions with Pb­(TFA)_4_ in HTFA. This demonstrates the enhanced reactivity of Pb­(IV) triflate complexes for methane oxidation compared to Pb­(IV) trifluoroacetate. The increased electron withdrawing ability of the triflate anion increases the Pb^2+/4+^ oxidation potential, resulting in a powerful Pb­(IV) oxidant, capable of C–H activation of CH_4_ in an electrophilic mechanism. Further studies with other alkanes are under way.

## Supplementary Material





## Notes

Materials and methods, synthetic procedures, NMR spectra and scXRD data (PDF)
(CCDC 2486641 to 2486644) (CIF)
