# Supplementary material for: Insights into Electrophilic Methane Activation with High-Valent Lead in Trifluoroacetic Acid and Trifluoromethanesulfonic Acid
Source: ACS Omega. 2026 Aug 11;11(33):50417–22. doi: 10.1021/acsomega.6c06786 (PMC13520021; doi:10.1021/acsomega.6c06786)
Supplement: Supplementary file 1 [file ao6c06786_si_001.pdf]

**Supporting Information**

# Insights into Electrophilic Methane Activation with High-valent Lead in Trifluoroacetic Acid and Trifluoromethanesulfonic Acid

Liam Parkin, Andrew J. P. White, Andrew Ashley* and George J. P. Britovsek*

Department of Chemistry, Imperial College London, Molecular Sciences Research Hub, White City Campus, 82 Wood Lane, London W12 0BZ, United Kingdom.
Corresponding author: g.britovsek@imperial.ac.uk

## Table of Contents

*General considerations* ... 2

*Synthesis and characterisation* ... 3

Synthesis of $Pb(TFA)_2(TFAH)_2$ ... 3

Synthesis of $Pb(OTf)_2(HOTf)$ ... 3

Alternative synthesis of $Pb(OTf)_2(HOTf)$ from $Pb(TFA)_2(HTFA)_2$ ... 4

Decomposition of $Pb(OTf)_4$ ... 4

Determination of Pb(IV) via iodometry ... 4

Synthesis of methyl(trifluoromethyl)sulfone $H_3CSO_2CF_3$ (MeTf) ... 5

Reaction of $Pb(OTf)_4$ with methane ... 5

*NMR Spectra* ... 6

*Cyclic voltammetry* ... 13

*X-Ray Crystallography* ... 14

The X-ray crystal structure of $Pb(TFA)_2(HTFA)_2$ ... 15

The X-ray crystal structure of $Pb(OTf)_2(HOTf)$-A ... 15

The X-ray crystal structure of $Pb(OTf)_2(HOTf)$-B ... 16

The X-ray crystal structure of $Pb(OTf)_2(HOTf)$-C ... 17

References ... 20

# General considerations

All reactions were conducted under a $N_2$ atmosphere, unless stated otherwise. All manipulations were carried out either in an MBraun Labmaster DP glovebox or by using standard Schlenk line techniques. Glassware was dried in an oven at 180 °C overnight before use. All solvents were degassed and dried before use: $CF_3SO_3H$, $CF_3COOH$ and $(CF_3CO)_2O$ were distilled prior to use; $(CF_3SO_2)_2O$ was used as supplied and stored in the glovebox. All other substrates were purchased from major suppliers: solids were dried under vacuum, while liquids were degassed and dried over 4 Å molecular sieves.

NMR spectra were recorded on Bruker AV-400 MHz, AV-500 MHz and DRX-400 spectrometers. $^{1}H$ NMR spectra were referenced internally to proteo solvent signals; $^{19}F$, NMR spectra were referenced externally to $CFCl_3$. For all solutions in NMR tubes that do not have a deuterated solvent, samples were shimmed manually, with no lock. High pressure NMR experiments were carried out in a Wilmad thin-walled high pressure NMR tube. Elemental analyses were carried out by the Elemental Analysis Service at London Metropolitan University. Electrochemical studies were carried out using a Metrohm Autolab PGSTAT302N potentiostat linked to a computer running Metrohm Autolab NOVA version 1.11 software, in conjunction with a three electrode cell comprising a glassy carbon disc working electrode, a platinum wire (99.99% purity) counter electrode, and a silver wire (99.99% purity) *pseudo*-reference electrode; all electrodes were polished with 0.3 µm α-alumina and dried prior to use. All electrochemical measurements were performed at ambient temperature under a dry $N_2$ atmosphere, in HOTf containing 0.1 M NaOTf as the supporting electrolyte and 0.5 mM of the analyte species of interest.

# Synthesis and characterisation

## Synthesis of $Pb(TFA)_2(TFAH)_2$

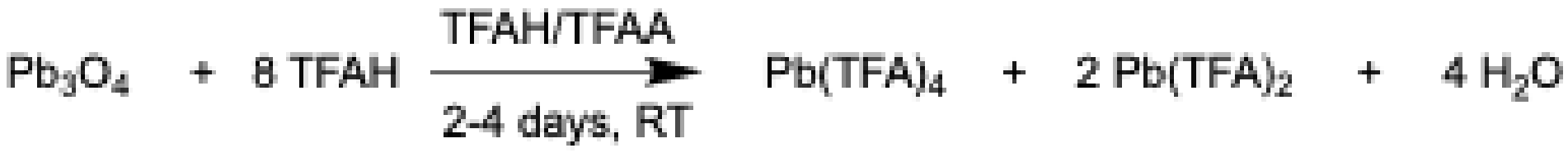


This synthesis was performed according to a literature procedure.[1]

$Pb_3O_4$ (0.6856 g, 1 mmol), TFAH (10 mL) and TFAA (3 mL, 22 mmol) were combined in an ampoule to give an orange suspension. This mixture was stirred at room temperature for 3 days. The orange suspension was filtered to give a colourless solution. The overall volume was reduced by half *in vacuo* and cooled (5 °C) overnight. Colourless needle shaped crystals formed which were suitable for single crystal XRD, showing that $Pb(TFA)_2(TFAH)_2$ had been crystallised (0.0750 g).

$^{19}$F NMR (377 MHz, $CDCl_3$) δ -74.55.

IR (FTIR) ν = 1595*b*, 1441*m*, 1154*b*, 855*s*, 792*s*, 725*s*, 695*s* $cm^{-1}$.

Elemental analysis found (calculated) for $Pb(TFA)_2(TFAH)_2$: C 11.93 (14.53); H 0.0 (0.30). Calculated for $Pb(TFA)_2$: C 11.09.

## Synthesis of $Pb(OTf)_2(HOTf)$

**Caution.** *Triflic acid is one of the strongest known protic acids. It is necessary to take adequate precautions to prevent contact with the skin and eyes. Precautions should also be taken to minimize inhalation of the corrosive vapours given off from the acid. Reactions with the neat acid must be conducted in a well-ventilated fume hood.*

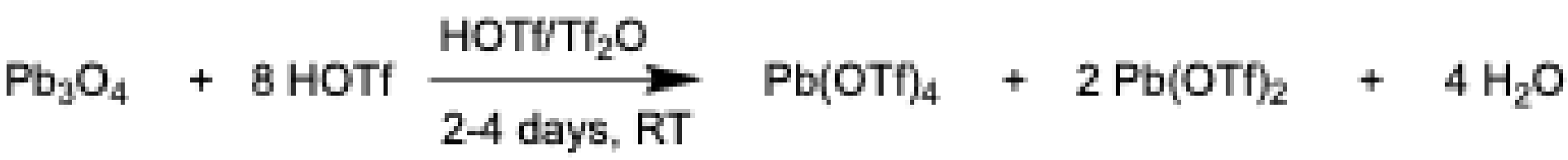


$Pb_3O_4$ (1.7542 g, 2.56 mmol), HOTf (10 mL), and $Tf_2O$ (3 mL, 18 mmol) were combined in an ampoule to give an orange suspension which was stirred at r.t. for 48 h. This gave a slightly yellow precipitate with a colourless solution. The solid was isolated by filtration and subsequently dried *in vacuo* (2.58 g, 77%). The filtrate was left at room temperature which afforded white crystals suitable for single crystal XRD that were characterised as $Pb(OTf)_2(HOTf)$–B.

$^{19}F$ NMR (377 MHz, $CD_3CN$) δ -79.16 ppm.

IR (FTIR) ν = 1204*b*, 1167*s*, 1025*m* $cm^{-1}$.

Elemental analysis found (calculated) for $Pb(OTf)_2(HOTf)$: C 5.55 (5.50); H 0.33 (0.15); S 13.29 (14.68). Calculated for $Pb(OTf)_2$: C 4.75; S 12.69.

## Alternative synthesis of $Pb(OTf)_2(HOTf)$ from $Pb(TFA)_2(HTFA)_2$

$Pb(TFA)_2(HTFA)_2$ (0.2403 g) and HOTf (5 mL) were combined in an ampoule. This was stirred at room temperature for 24 h. After this time the volume was reduced by half in vacuo and a small portion of the solution was taken and placed inside a small vial, which itself was in a large vial that contained $CHCl_3$, and a vapour diffusion was performed. Crystals suitable for XRD were obtained which gave structure $Pb(OTf)_2(HOTf)$-A.

## Decomposition of $Pb(OTf)_4$

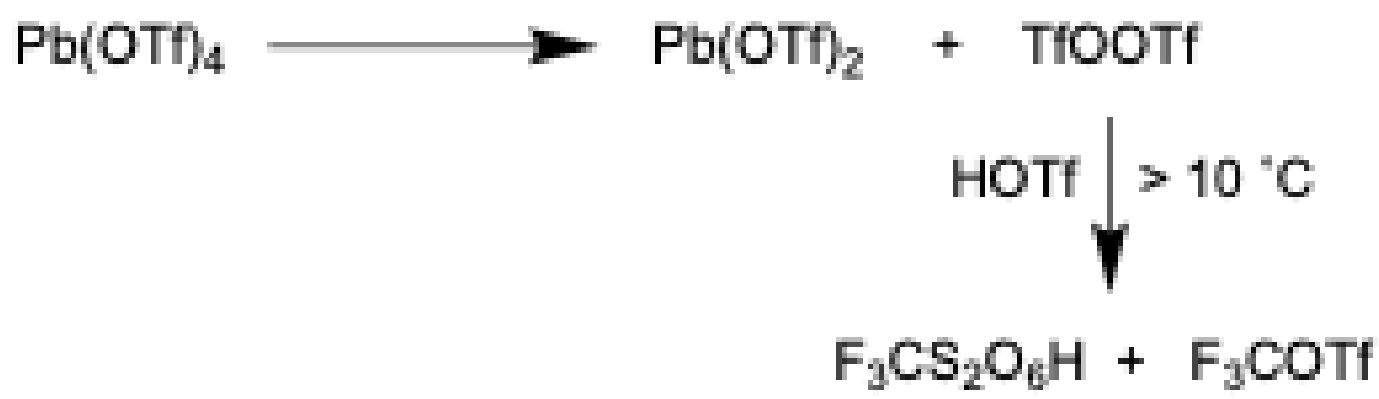


Formation of $F_3COTf$ was observed by $^{19}F$ NMR (see Figure S2):

$^{19}F$ NMR (377 MHz, HOTf/$C_6D_6$) δ -54.45 (q, $^5J_{F\text{-}F}$ = 3.5 Hz, $CF_3OTf$), -75.09 (q, $^5J_{F\text{-}F}$ = 3.6 Hz, $CF_3OSO_2CF_3$). Data compares with literature.[2]

The reaction of $F_3CSO_3H$ and $SO_3$ to give $F_3CS_2O_6H$ has been reported previously.[3]

## Determination of Pb(IV) via iodometry.[4]

A sample of $Pb(OTf)_4/2Pb(OTf)_2$ (M = 1814.20, 53.7 mg), obtained from the reaction of $Pb_3O_4$ in HOTf/$Tf_2O$, contains 53.7 mg/1814 g/mol = 0.03 mmol Pb(IV) in theory. This was treated with an excess of KI (100 mg) and dissolved in 50 mL water, saturated with NaOAc and starch. The amount of $I_2$ generated was titrated with $Na_2S_2O_3$ (0.1 M) and 0.2 mL was required, which is 0.02 mmol. This corresponds to 0.01 mmol active Pb(IV) in solution or 33 % of the theoretical amount based on $Pb(OTf)_4/2Pb(OTf)_2$.

## Synthesis of methyl(trifluoromethyl)sulfone $H_3CSO_2CF_3$ (MeTf)

Adapted from a literature synthesis.[5] $Tf_2O$ (3 g) was added to diethyl ether (50 mL). This was cooled to -78 °C and MeMgBr (3 M, $Et_2O$, 3.54 mL) was added dropwise. This was allowed to slowly warm to room temperature over 2 h. Sat. $NH_4Cl$ (aq.) was added and the organic layer collected. The aqueous layer was washed with DCM (3 x 10 mL) and the organic layers were collected and combined. The volatiles were removed in vacuo. The product was distilled (bulb to bulb) to give MeTf.

$^1$H NMR (400 MHz, $CDCl_3$) δ 3.14 (q, $^4J_{HF}$ = 0.9 Hz, 1H).

$^{19}$F NMR (377 MHz, $CDCl_3$) δ -79.82.

## Reaction of $Pb(OTf)_4$ with methane

A mixture of $Pb(OTf)_2$/$Pb(OTf)_4$ (100 mg) generated from $Pb_3O_4$ and HOTf, (containing 33 mol% active Pb(IV) in solution) and HOTf (0.5 mL) were combined in a Wilmad HP NMR tube. The NMR tube was pressurised with $CH_4$ to 5 bar. The solution was heated for the desired amount of time and temperature and the reaction progress was monitored by $^1$H-NMR (Figure S3). For the reaction at 60 ˚C, the amount of MeOTf and MeTf after 15 hours were 2% and 3.2%, respectively (determined by NMR integration against the solvent signal and based on the amount of active Pb(IV) oxidant in solution).

# NMR Spectra

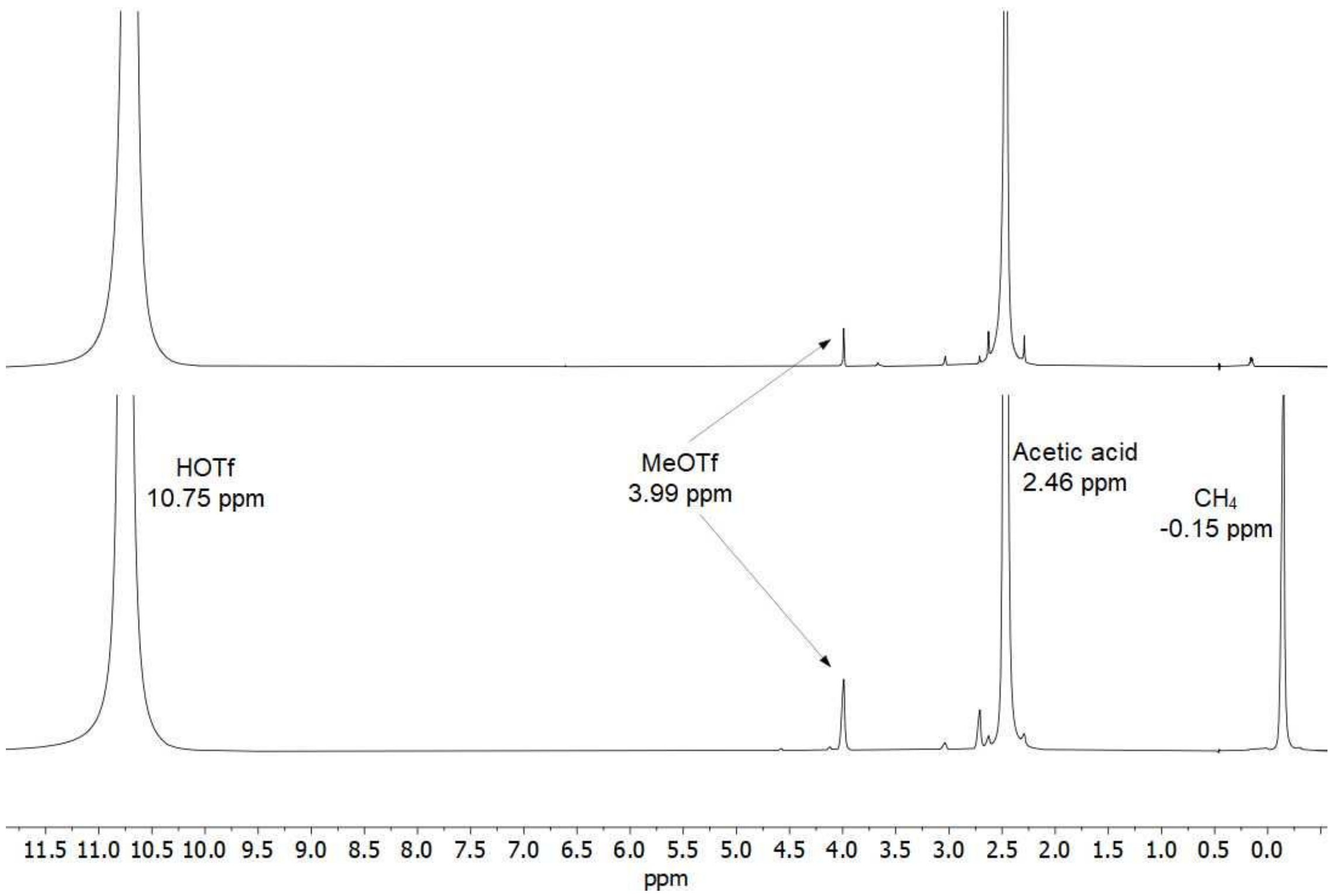


Figure S1. Reaction of $Pb(OAc)_4$ in HOTf (solvent) heated at 50 °C for 15 h. Above: $^1H$ NMR spectrum reaction performed under $N_2$. Below: $^1H$ NMR spectrum of reaction performed under 6 bar $CH_4$. MeOTf production is observed regardless of the addition of methane, presumably due to decarboxylation of AcOOTf to MeOTf.

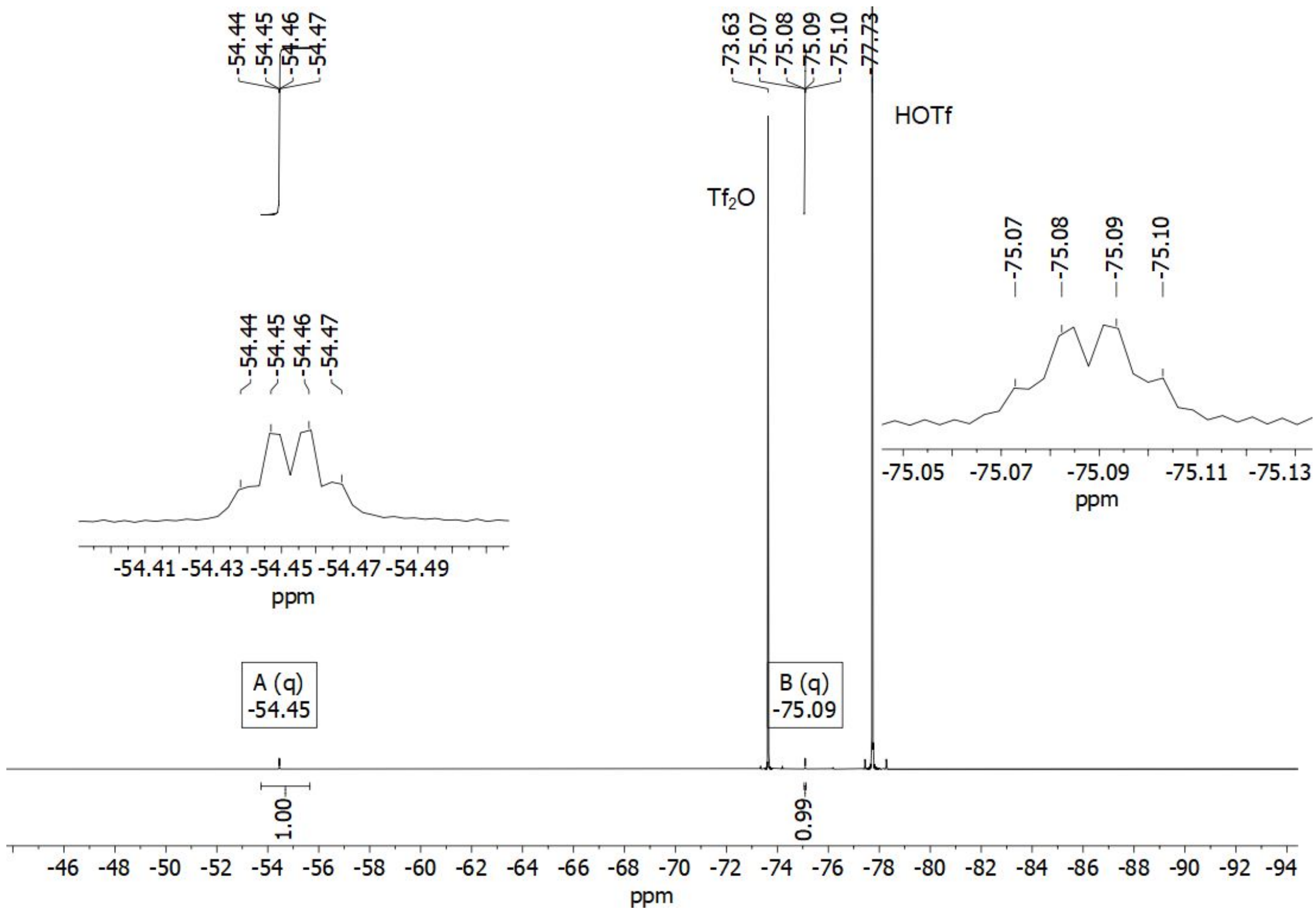


Figure S2. $^{19}F\{^1H\}$ NMR spectrum of the reaction of $Pb_3O_4$ (50 mg) in HOTf (0.3 mL) and $Tf_2O$ (0.2 mL) at room temperature after 17 h, showing formation of $F_3COTf$.

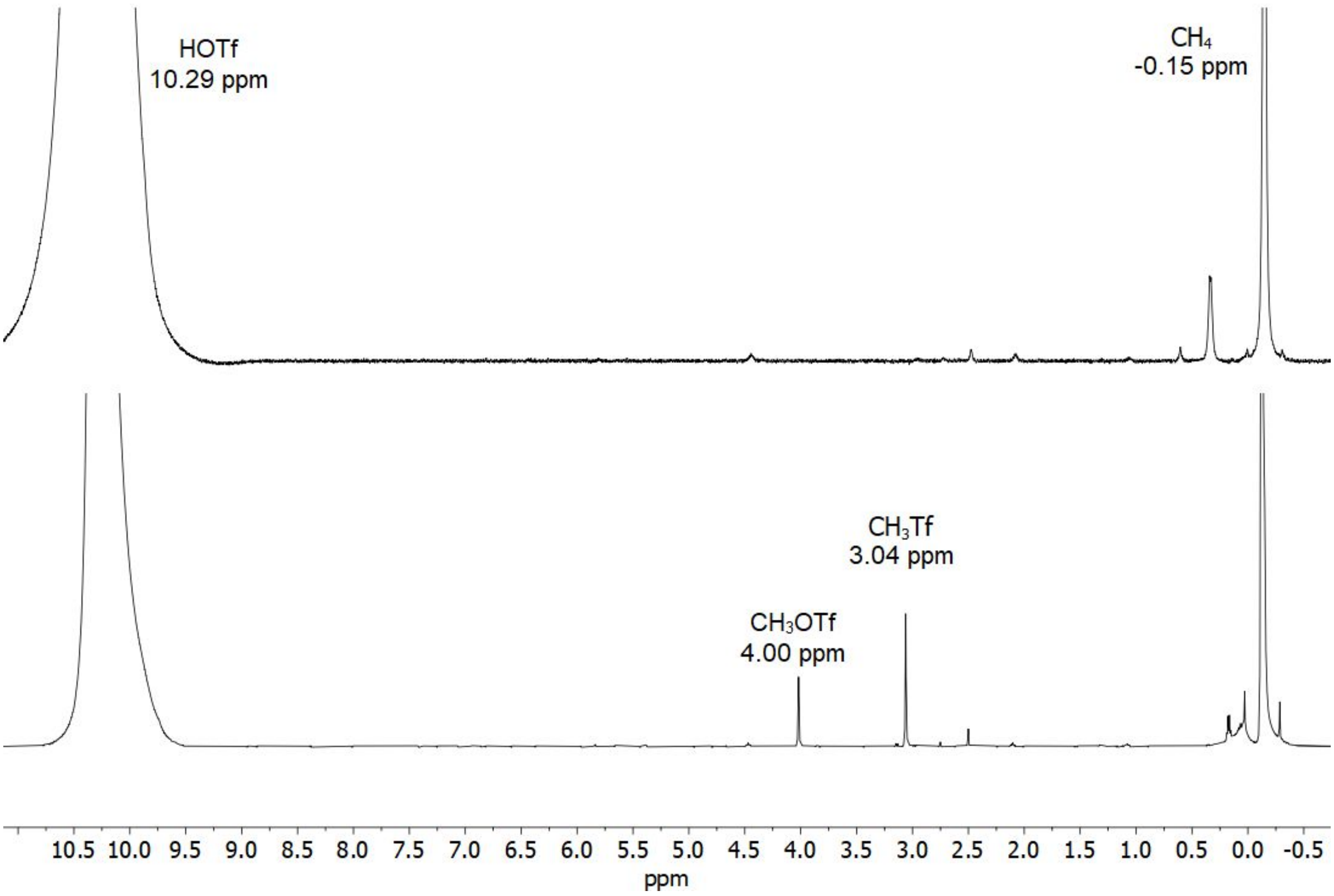


Figure S3. $^1$H-NMR spectra of the reaction of $Pb(OTf)_4$ (generated from $Pb_3O_4$) in $HOTf/Tf_2O$ (0.5 mL) + 5 bar $CH_4$ heated at 60 °C. Above: at t = 0 h. Below: after t = 15 h.

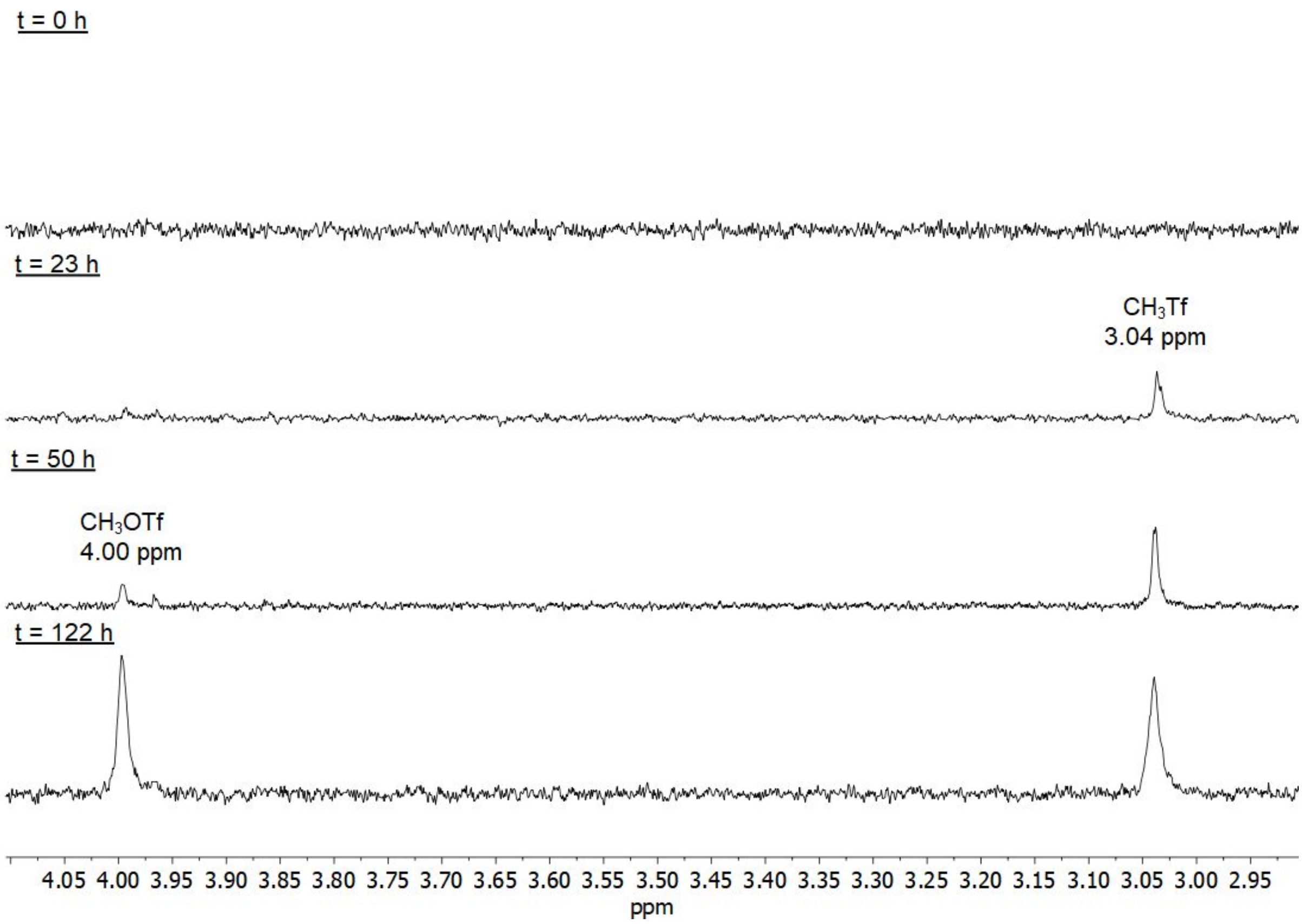


Figure S4. Stacked $^1$H-NMR spectra of methane activation with $Pb(OTf)_4$ in HOTf/$Tf_2O$ (prepared from $Pb_3O_4$) at 298K monitored over time. MeOTf (4.00 ppm) and MeTf (3.04 ppm,$^4J_{HF}$ = 0.9 Hz) increase in concentration as time increases. $CH_4$ pressure = 5 bar.

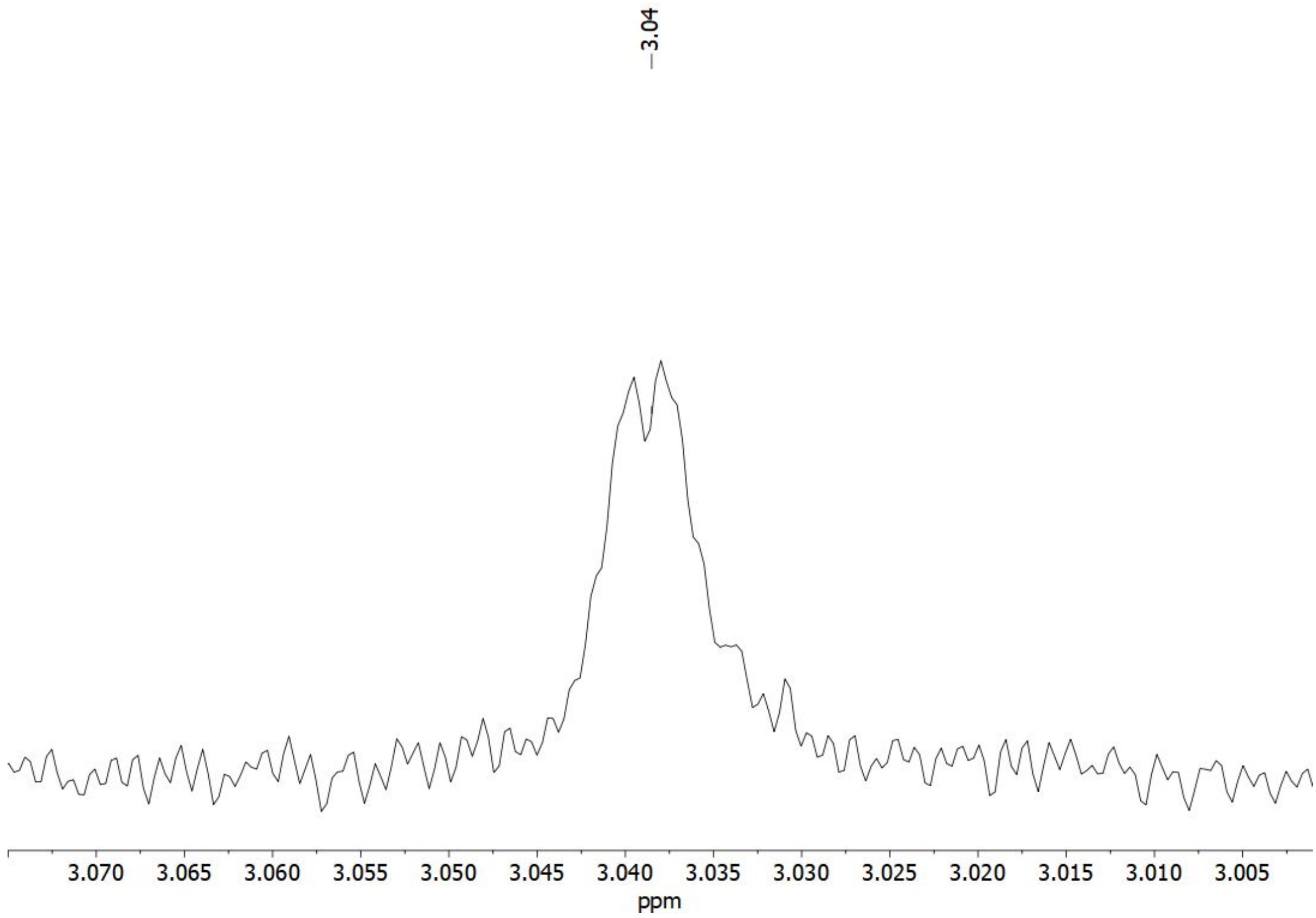


Figure S5. Expansion of the $^{1}$H-NMR spectrum in Figure S4, showing MeTf with $^{4}J_{F\text{-}H}$ = 0.9 Hz.

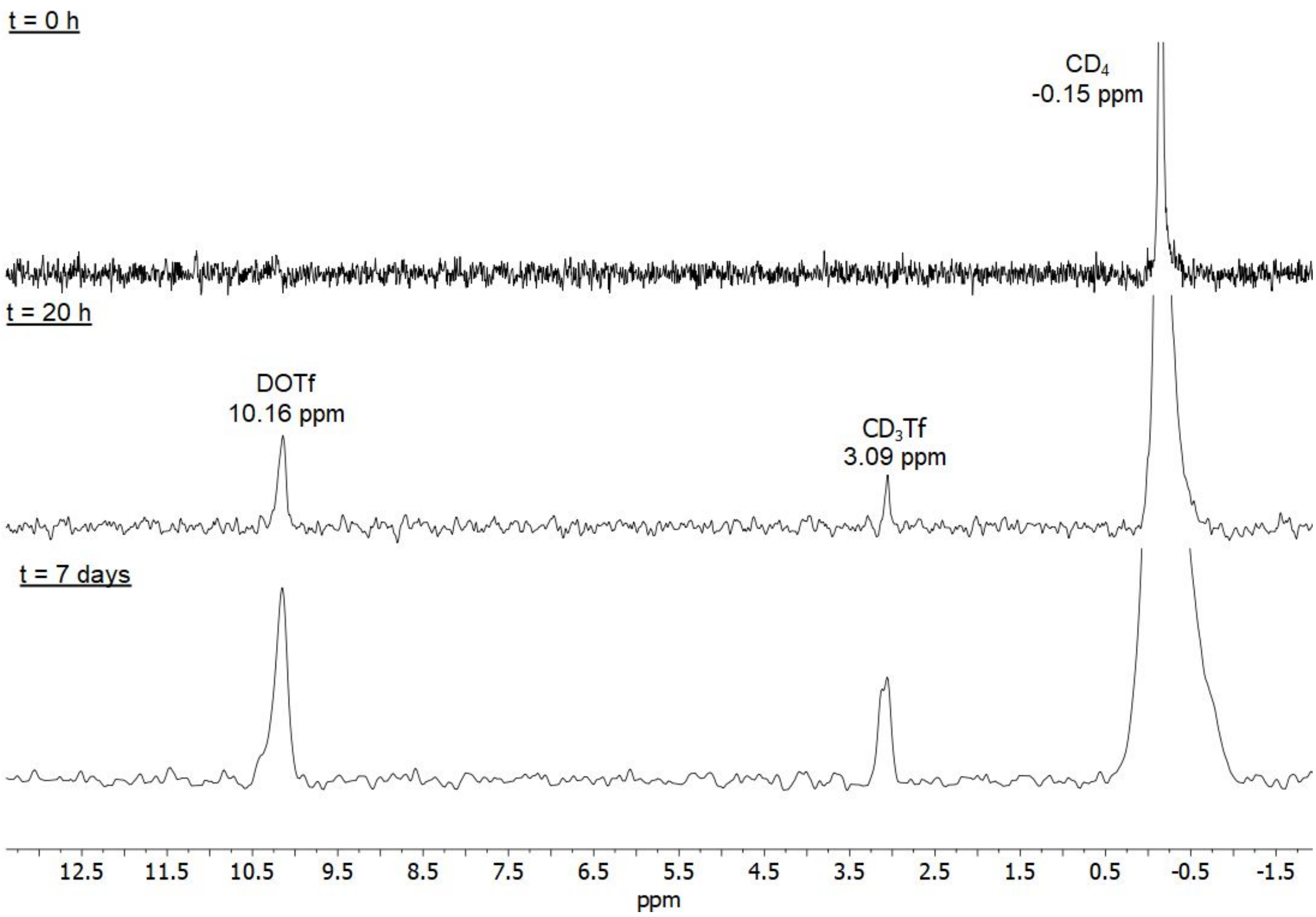


Figure S6. Stacked $^2$H-NMR spectra of $CD_4$ oxidation in HOTf/$Tf_2O$ using $Pb(OTf)_4$ (generated from $Pb_3O_4$ and HOTf) and 6 bar $CD_4$, heated at 60 °C, showing formation of $CD_3Tf$ but no $CD_3OTf$ is observable after 7 days.

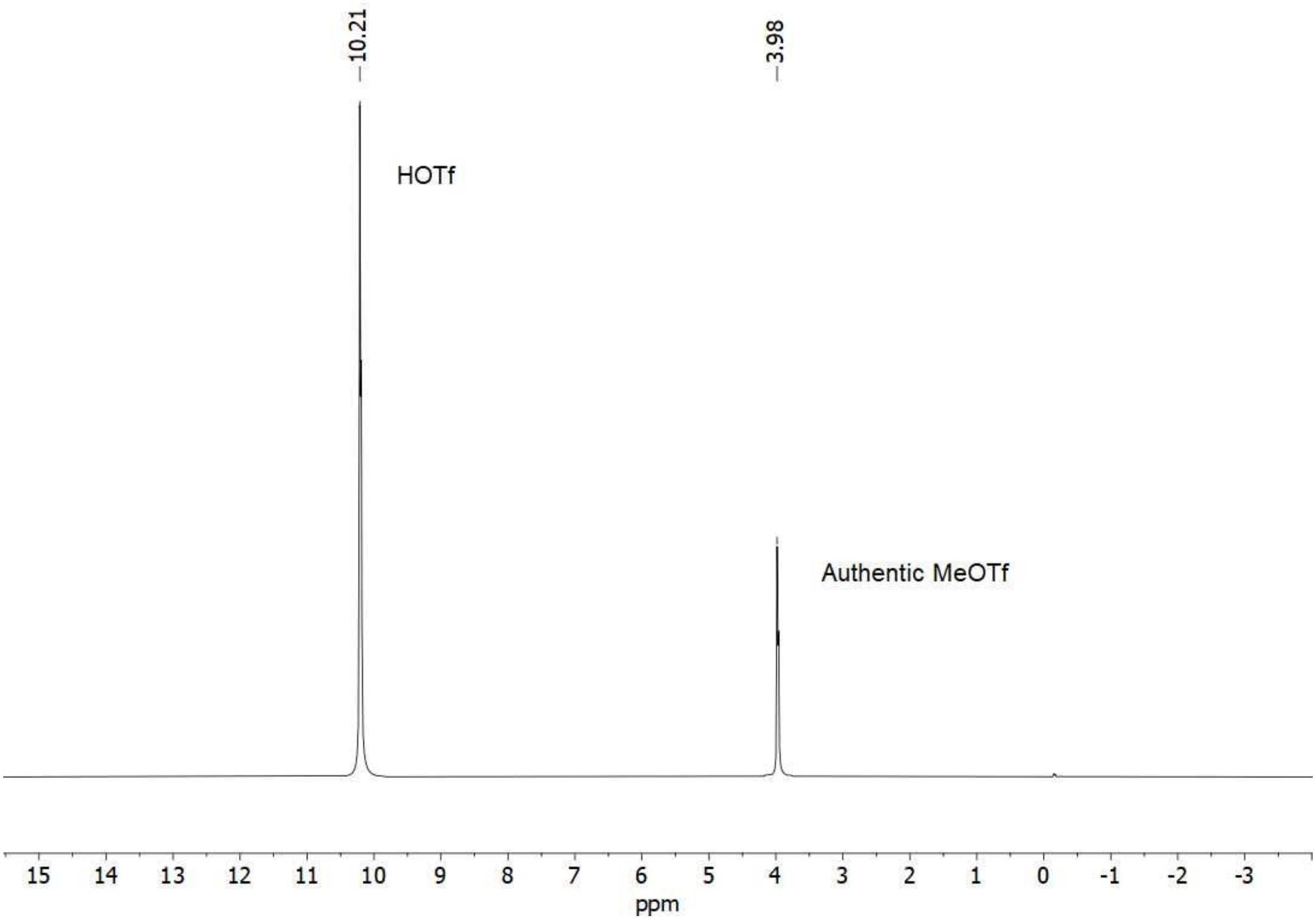


Figure S7: $^{1}H$ NMR reference spectrum of MeOTf in HOTf (298K).

# Cyclic voltammetry

Electrochemical experiments for $Pb(OTf)_2$, prepared according to literature,[6] using cyclic voltammetry were carried out at room temperature in neat HOTf (5 mL), using NaOTf (0.1 M) as electrolyte.[7] All experiments were performed inside an MBraun Labmaster 130 glovebox, under nitrogen, using a glassy carbon working electrode (5 mm), a Pt wire counter electrode and an Ag wire as a quasi-reference electrode. A solution of $[Ru(bipy)_3]Cl_2$ (0.5 mM) in HOTf was used as an external standard and the $Ru^{2+/3+}$ redox couple was observed at +0.31 V (vs. Ag, peak separation 140 mV) as shown in Figure S8. This value compares well with the value of +0.32 V (vs. Ag) reported previously.[7]

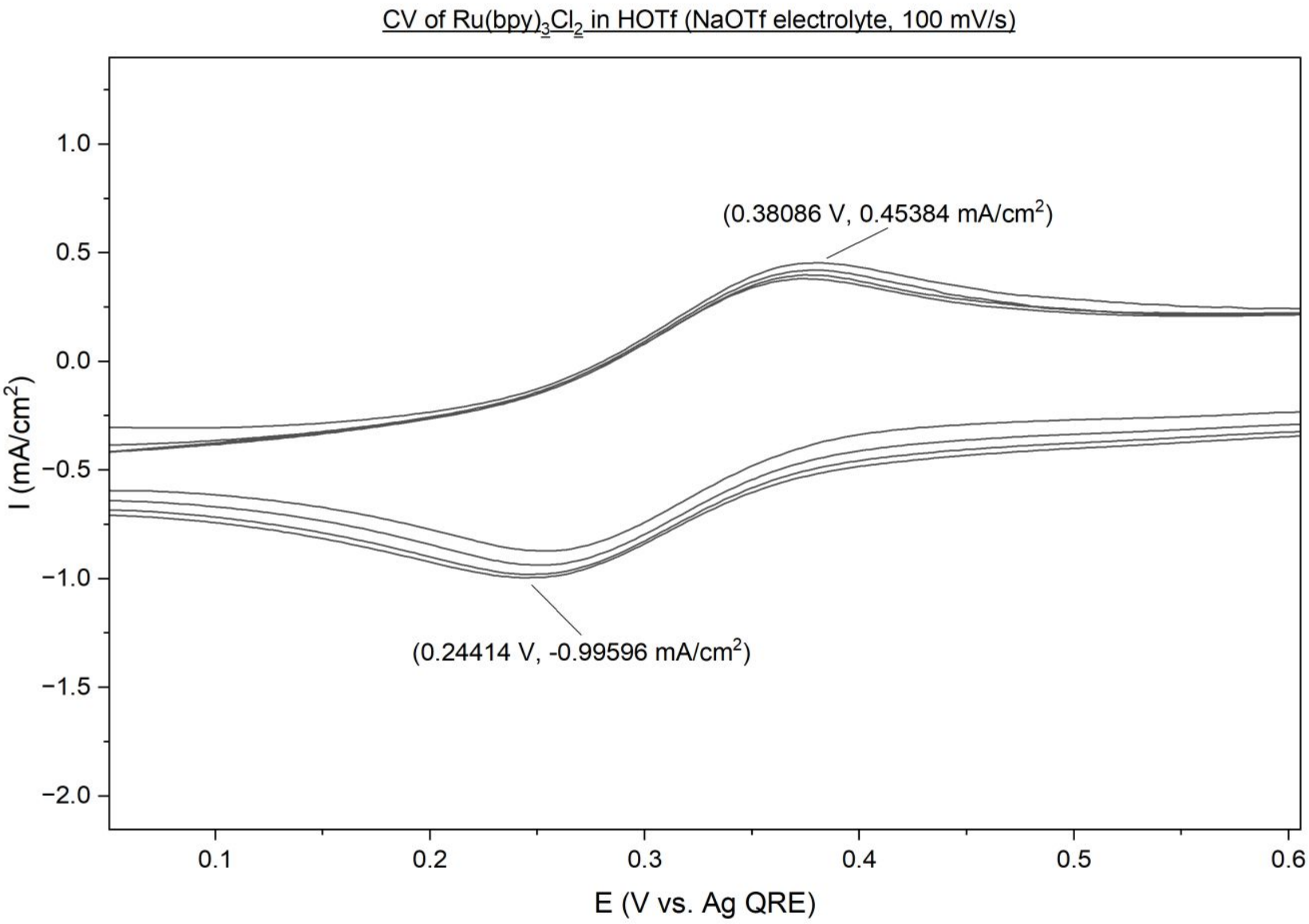


Figure S8. Cyclic voltammogram of $[Ru(bipy)_3]Cl_2$ (0.5 mM) in HOTf with NaOTf (0.1 M) as electrolyte.

# X-Ray Crystallography

**Table S1**. Crystal Data, Data Collection and Refinement Parameters for the structures of **Pb(TFA)$_2$(HTFA)$_2$**, **Pb(OTf)$_2$(HOTf)-A**, **Pb(OTf)$_2$(HOTf)-B** and **Pb(OTf)$_2$(HOTf)-C**.

| data | Pb(TFA)$_2$(HTFA)$_2$ | Pb(OTf)$_2$(HOTf)-A | Pb(OTf)$_2$(HOTf)-B | Pb(OTf)$_2$(HOTf)-C |
|---|---|---|---|---|
| **formula** | $C_8H_2F_{12}O_8Pb$ | $C_3HF_9O_9PbS_3$ | $C_3HF_9O_9PbS_3$ | $C_3HF_9O_9PbS_3$ |
| **formula weight** | 661.29 | 655.41 | 655.41 | 655.41 |
| **colour, habit** | colourless -- | colourless needles | colourless -- | colourless -- |
| **temperature / K** | 173 | 173 | 173 | 173 |
| **crystal system** | monoclinic | triclinic | triclinic | triclinic |
| **space group** | $P2_1/c$ (no. 14) | $P$-1 (no. 2) | $P$-1 (no. 2) | $P$-1 (no. 2) |
| ***a* / Å** | 5.60864(14) | 4.9487(2) | 4.9419(2) | 4.9390(4) |
| ***b* / Å** | 23.8236(3) | 11.2370(6) | 11.2112(7) | 11.2135(8) |
| ***c* / Å** | 12.2797(2) | 13.3873(3) | 13.3658(9) | 13.3792(9) |
| **α / deg** | 90 | 97.280(3) | 97.262(5) | 97.237(6) |
| **β / deg** | 99.042(2) | 99.551(3) | 99.568(5) | 99.587(6) |
| **γ / deg** | 90 | 102.409(4) | 102.400(5) | 102.344(6) |
| **$V$ / Å$^3$** | 1620.40(5) | 706.72(5) | 703.01(7) | 703.63(9) |
| ***Z*** | 4 | 2 | 2 | 2 |
| **$D_c$ / g cm$^{-3}$** | 2.711 | 3.080 | 3.096 | 3.089 |
| **radiation used** | Cu-Kα | Mo-Kα | Mo-Kα | Cu-Kα |
| **μ / mm$^{-1}$** | 21.952 | 12.533 | 12.599 | 29.049 |
| **no. of unique reflns** | | | | |
| **measured ($R_{int}$)** | 3253 (0.0824) | 5928 (0.0505) | 2843 (0.0690) | 2632 (0.0365) |
| **obs, $\|F_o\| > 4\sigma(\|F_o\|)$** | 2735 | 4493 | 2496 | 2367 |
| **completeness (%)** [a] | 100 | 99.8 | 96.5 | 97.4 |
| **no. of variables** | 303 | 232 | 231 | 230 |
| **$R_1$(obs), $wR_2$(all)** [b] | 0.0408, 0.0835 | 0.0426, 0.1328 | 0.0672, 0.1777 | 0.0352, 0.0894 |
| **CCDC code** | 2486641 | 2486642 | 2486643 | 2486644 |

[a] Completeness to 0.84 Å resolution. [b] $R_1 = \Sigma||F_o| - |F_c||/\Sigma|F_o|$; $wR_2 = \{\Sigma[w(F_o^2 - F_c^2)^2] / \Sigma[w(F_o^2)^2]\}^{1/2}$; $w^{-1} = \sigma^2(F_o^2) + (aP)^2 + bP$.

Table S1 provides a summary of the crystallographic data for the structures of **$Pb(TFA)_2(HTFA)_2$**, **$Pb(OTf)_2(HOTf)$-A**, **$Pb(OTf)_2(HOTf)$-B** and **$Pb(OTf)_2(HOTf)$-C**. The lead precursors for the different structures of **$Pb(OTf)_2(HOTf)$** are as follows:

**$Pb(OTf)_2(HOTf)$-A: from $Pb(TFA)_2(HTFA)_2$**

**$Pb(OTf)_2(HOTf)$-B: from $Pb_3O_4$**

**$Pb(OTf)_2(HOTf)$-C: from $Pb(OAc)_4$**

Data were collected using Agilent Xcalibur PX Ultra A [**$Pb(TFA)_2(HTFA)_2$**, and **$Pb(OTf)_2(HOTf)$-C**] and Xcalibur 3 E [**$Pb(OTf)_2(HOTf)$-A** and **$Pb(OTf)_2(HOTf)$-B**] diffractometers, and the structures were solved and refined using the OLEX2,[8] SHELXTL[9] and SHELX-2013[10] program systems. CCDC 2486641 to 2486644.

## The X-ray crystal structure of $Pb(TFA)_2(HTFA)_2$

The C14- and C34-based trifluoromethyl groups in the structure of **$Pb(TFA)_2(HTFA)_2$** were both found to be disordered. For the former two orientations were identified of *ca*. 66 and 34% occupancy, whilst for the latter three orientations were identified of *ca*. 38, 37 and 24% occupancy. The geometries of each set of orientations were optimised, the thermal parameters of adjacent atoms were restrained to be similar, and only the non-hydrogen atoms of the 66% occupancy orientation of the C14-based group were refined anisotropically (all the other partial occupancy atoms were refined isotropically). The O–H hydrogen atoms on O13 and O33 could not be located from $\Delta F$ maps and so were initially added using the SHELX HFIX 147 command and then that command was removed and they were refined freely subject to an O–H distance constraint of 0.90 Å.

## The X-ray crystal structure of $Pb(OTf)_2(HOTf)$-A

The crystal of **$Pb(OTf)_2(HOTf)$-A** that was studied was found to be a two component twin in a *ca*. 62:38 ratio, with the two lattices related by the approximate twin law [0.99 –0.02 0.00 0.09 1.01 0.01 –0.01 0.00 1.00]. This is likely to be the main factor in the somewhat high residual electron density of *ca*. 3.0 $eÅ^{-3}$ (see the discussion of **$Pb(OTf)_2(HOTf)$-B** below). The O–H hydrogen atom on O3 in the structure of **$Pb(OTf)_2(HOTf)$-A** could not be located from a $\Delta F$ map and so was initially added using the SHELX HFIX 147 command and then that command was removed and it was refined freely subject to an O–H distance constraint of 0.90 Å.

## The X-ray crystal structure of $Pb(OTf)_2(HOTf)$-B

The crystal of **$Pb(OTf)_2(HOTf)$-B** that was studied was found to be a two component multi-crystal, and the structure presented here comes from component 1 (component 2 was not solved). In terms of indexing, component 1 uses *ca*. 72% of the observed spots (62% separate, 9% overlapped) whilst component 2 uses *ca*. 26% of the observed spots (17% separate, 9% overlapped) with *ca*. 12% unused.

Though the structure of component 2 could not be solved, processing the data as a multi-crystal including component 2 allowed for a much improved data set for the major component 1 compared to processing the data as component 1 only (*i.e.* without acknowledging the multi-crystal). For example, the maximum peak/hole reduces from *ca*. 10.6/–2.6 to 5.9/–3.8 $eÅ^{-3}$. However, whilst a clear improvement, this is still both a very high maximum residual electron density, and a high background noise level. That this is due to the multi-crystal can be seen by a comparison with the two other determinations of the structure of **$Pb(OTf)_2(HOTf)$** presented here – in **$Pb(OTf)_2(HOTf)$-A** the crystal was modelled as a two component twin with maximum peak/hole of *ca*. 3.0/–1.2 $eÅ^{-3}$, whilst in **$Pb(OTf)_2(HOTf)$-C** the crystal showed no signs of being a twin or a multi-crystal, and even though it was the only one of the three where the data was collected using Cu-Kα radiation (and would therefore be expected to have largest absorption effects) the maximum peak/hole were the lowest of the three structures at *ca*. 1.9/–1.6 $eÅ^{-3}$. This pattern strongly suggests that the crystal quality (and thus the intensity data) is the chief factor in the poor residual electron density seen here rather than any defect in the model.

The O–H hydrogen atom on O3 in the structure of **$Pb(OTf)_2(HOTf)$-B** could not be located from $\Delta F$ maps, and adding it using the SHELX HFIX 147 command had it move into the wrong place in subsequent refinements (as compared to the same atom in the better resolved structures **$Pb(OTf)_2(HOTf)$-A** and **$Pb(OTf)_2(HOTf)$-C**). So here it was added in the same position as seen in **$Pb(OTf)_2(HOTf)$-C** (by the simple method of copying the coordinates) then it was refined freely subject to an O–H distance constraint of 0.90 Å. This kept it pointing towards the evident hydrogen bonding acceptor (O22 in an adjacent molecule) though with a very large thermal parameter. This less than ideal behaviour is not exactly a surprise given the problems with this data set detailed above.

The information we have on component 2 is sparse. We have a unit cell [$a$ = 12.887(2), $b$ = 4.9992(6), $c$ = 15.381(3) Å, α = 90.170(12), β = 99.135(15), γ = 90.050(13)°, $V$ = 978.4(3) $Å^3$] which is potentially body-centred monoclinic but with the variance from ideal of the alpha

angle and no solved structure, we cannot be certain that this symmetry is correct. The volume is *ca*. 275 Å$^3$ larger than component 1, so approximately 20 extra non-hydrogen atoms in the unit cell (based on component 1 having 50 non-hydrogen atoms in a volume of 703 Å$^3$, so about 14 Å$^3$ per non-hydrogen atom—this assumes that component 2 is a chemically similar species). There is some indication that it might be another lead-containing species (we can get a less than terrible *R*-factor with just a couple of lead atoms) but we could not build anything that looks at all chemically sensible (perhaps in part because of wrong symmetry). Dropping down to space group *P*1 to avoid this did not help. From a chemical standpoint the most likely candidate seems to be $Pb(OTf)_2(HOTf)_2$, in analogy to $Pb(TFA)_2(HTFA)_2$, which has an extra triflic acid molecule compared to component 1. This is 8 non-hydrogen atoms more per metal centre, and so 16 extra non-hydrogen atoms per unit cell compared to the 20 estimated above for the observed larger unit cell volume.

## The X-ray crystal structure of $Pb(OTf)_2(HOTf)$-C

Unlike the crystals used for the structural determinations of **$Pb(OTf)_2(HOTf)$-A** and **$Pb(OTf)_2(HOTf)$-B**, the crystal used for the analysis of **$Pb(OTf)_2(HOTf)$-C** showed no signs of any twinning/multi-crystal and instead appeared to be entirely single, and thus unsurprisingly gives the highest quality final structure out of the three (see the discussion of **$Pb(OTf)_2(HOTf)$-B** above). The O–H hydrogen atom on O3 in the structure of **$Pb(OTf)_2(HOTf)$-C** could not be located from a Δ*F* map and so was initially added using the SHELX HFIX 147 command and then that command was removed and it was refined freely subject to an O–H distance constraint of 0.90 Å.

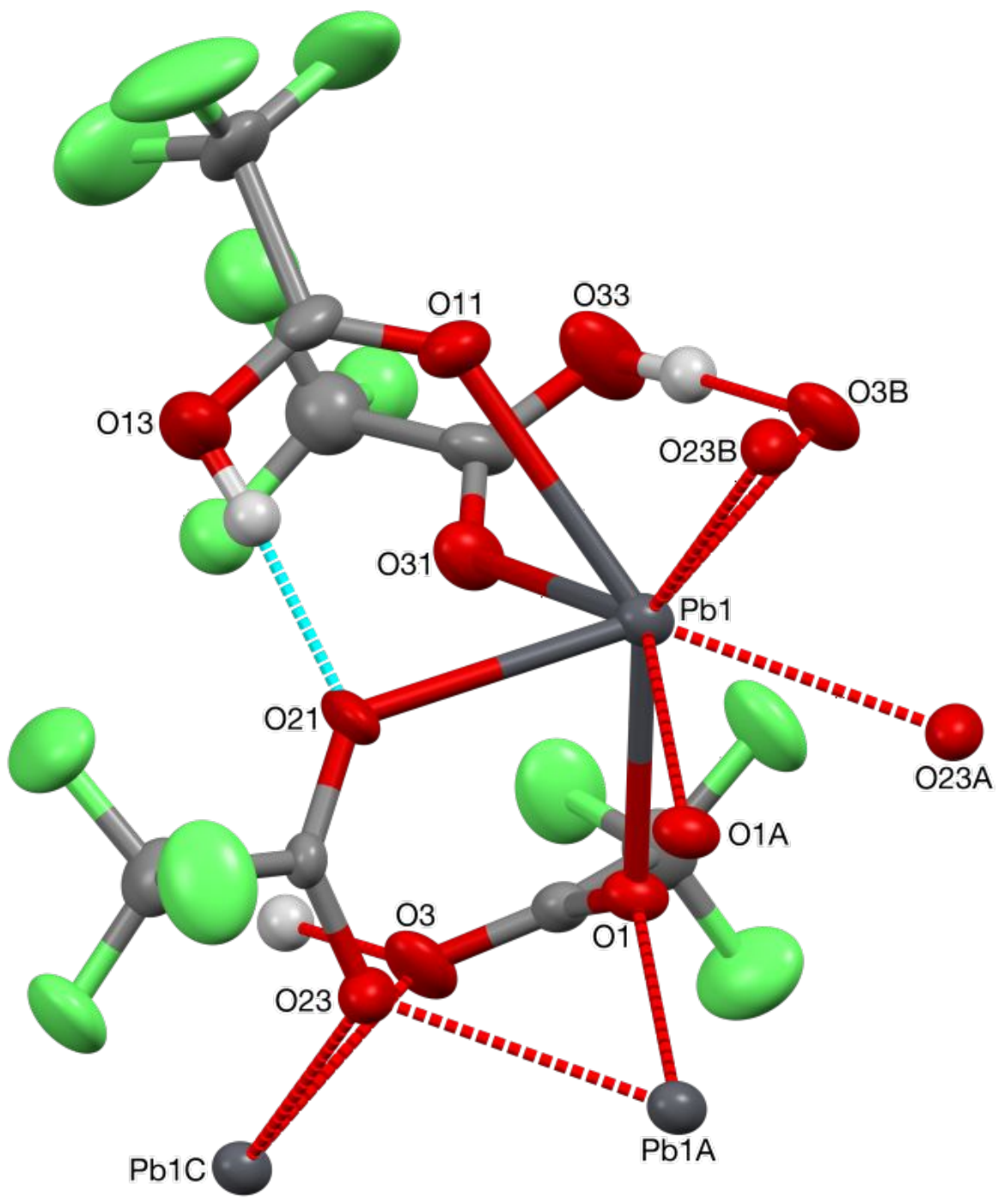


**Figure S10.** The crystal structure of **Pb(TFA)$_2$(HTFA)$_2$** showing the asymmetric unit and how it links to adjacent atoms (50% probability ellipsoids).

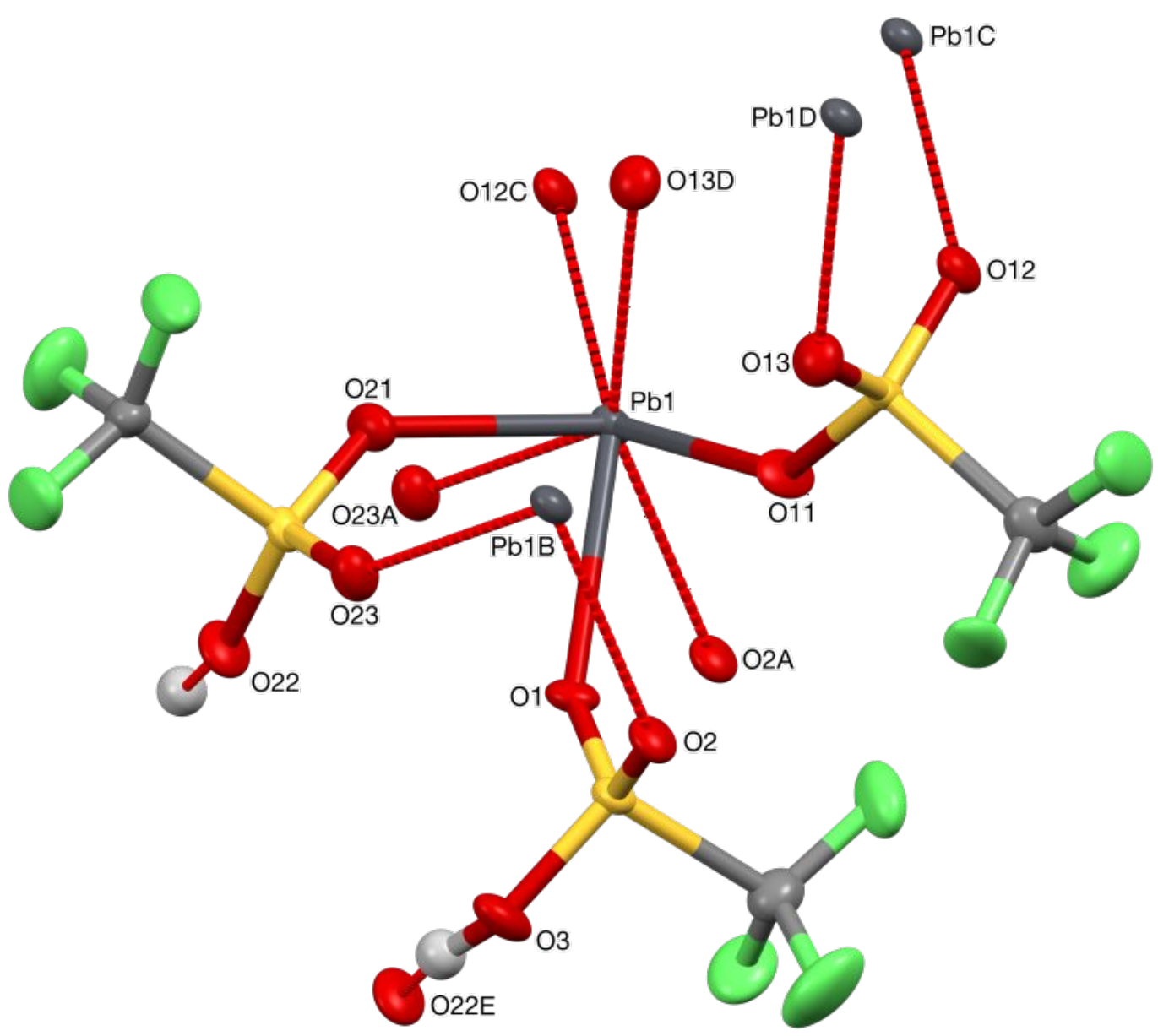


**Figure S11.** The crystal structure of **Pb(OTf)$_2$(HOTf)-A** showing the asymmetric unit and how it links to adjacent atoms (50% probability ellipsoids).

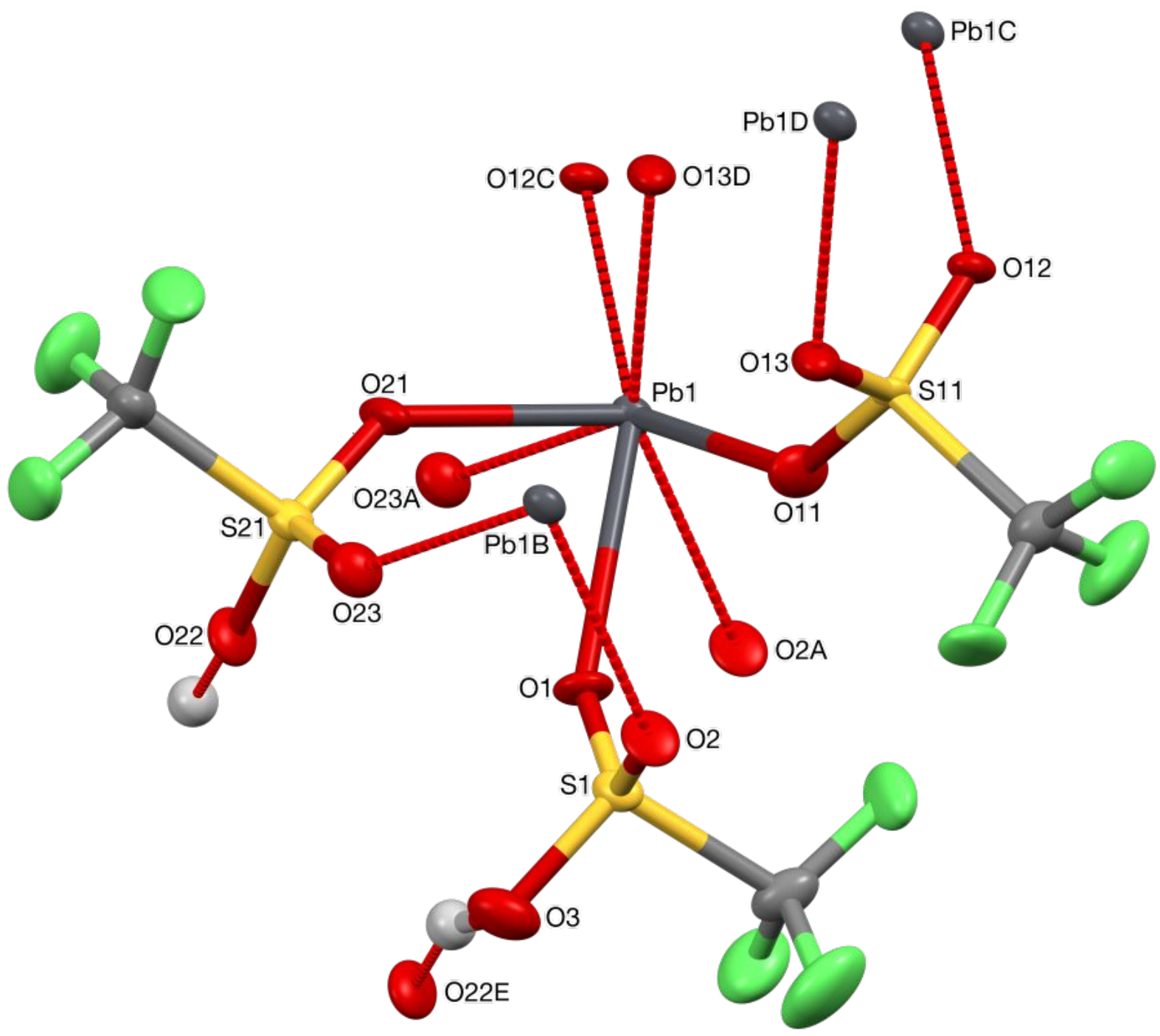


**Figure S12.** The crystal structure of **Pb(OTf)$_2$(HOTf)-B** showing the asymmetric unit and how it links to adjacent atoms (50% probability ellipsoids).

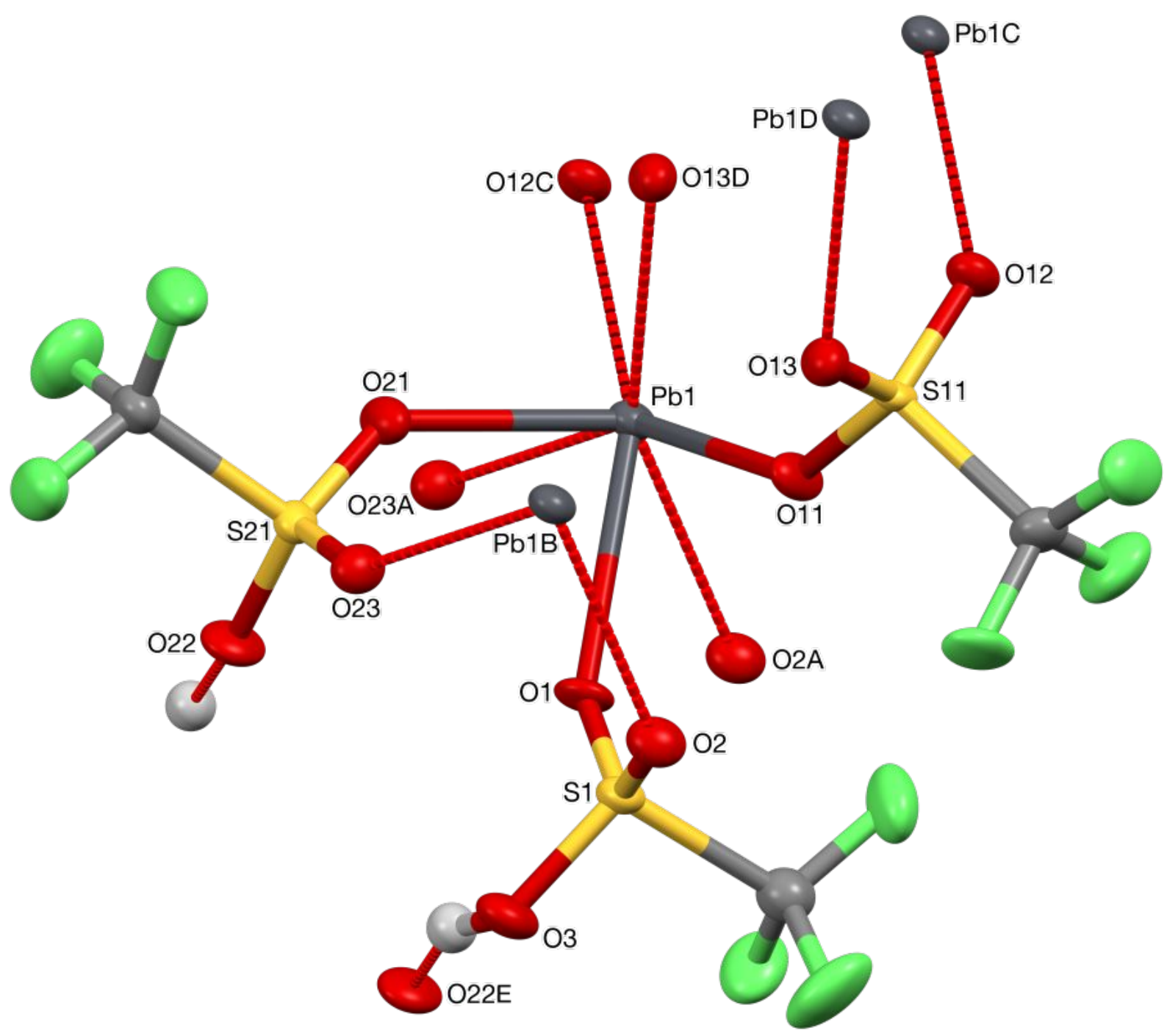


**Figure S13.** The crystal structure of **Pb(OTf)$_2$(HOTf)-C** showing the asymmetric unit and how it links to adjacent atoms (50% probability ellipsoids).

# References


1. Bell, H. C.; Kalman, J. R.; Pinhey, J. T.; Sternhell, S., The Chemistry of Aryllead (IV) Tricarboxylates. Synthesis. *Aus. J. Chem.* **1979,** *32*, 1521-1530.

2. Noftle, R. E.; Cady, G. H., Preparation and Properties of Bis(trifluoromethylsulfuryl) Peroxide and Trifluoromethyl Trifluoromethanesulfonate. *Inorg. Chem.* **1965,** *4*, 1010-1012.

3. Brunel, D.; Oudrhiri-Hassani, M.; Choukroun, H.; Germain, A., Oxydation anodique des acides perfluoroalcanesulfoniques. P (a) nic et mécanisme de formation des esters sulfoniques et pyrosulfoniques, et des perfluoroalcanes. *Nouveau Journal de Chimie* **1984,** *8*, 99-106.

4. Khenkin, A. M.; Herman, A.; Haviv, E.; Neumann, R., Electrocatalytic Oxyesterification of Hydrocarbons by Tetravalent Lead. *ACS Catalysis* **2021,** *11*, 10494-10501.

5. Kuang, Y.; Zhang, T.; Lu, Q.; Fu, Z.; Wu, Y.; Huang, J.; Zhao, J. Preparation method of trifluoromethyl alkyl sulfone. CN115304521, 2022.

6. Bergstroem, P.-A.; Frech, R., Raman and Infrared Study of the Polymer Electrolytes Formed by Poly(propylene oxide) with $Sn(CF_3SO_3)_2$ and $Pb(CF_3SO_3)_2$. *J. Phys. Chem.* **2002,** *99*, 12603-12611.

7. Bernhard, P.; Diab, H.; Ludi, A., Cyclic voltammetry in trifluoromethanesulfonic acid: facile electrochemistry up to +3 V. *Inorg. Chim. Acta* **1990,** *173*, 65-68.

8. O.V. Dolomanov, L.J. Bourhis, R.J. Gildea, J.A.K. Howard, H. Puschmann, *J. Appl. Cryst.*, **2009**, *42*, 339-341.

9. SHELXTL v5.1, Bruker AXS, Madison, WI, 1998.

10. SHELX-2013, G.M. Sheldrick, *Acta Cryst.*, **2015**, *C71*, 3-8.
